# Enrichment and proteomic identification of *Cryptosporidium parvum* oocyst wall

**DOI:** 10.1186/s13071-022-05448-8

**Published:** 2022-09-23

**Authors:** Luyang Wang, Yuexin Wang, Zhaohui Cui, Dongfang Li, Xiaoying Li, Sumei Zhang, Longxian Zhang

**Affiliations:** 1 College of Veterinary Medicine, Henan Agricultural University, Zhengzhou, 450002 China; 2 International Joint Research Center of National Animal Immunology, Zhengzhou, 450046 China; 3 Key Laboratory of Quality and Safety Control of Poultry Products (Zhengzhou), Ministry of Agriculture and Rural Affairs, Zhengzhou, People’s Republic of China

**Keywords:** *Cryptosporidium parvum*, Oocyst wall, Proteomics, Detection

## Abstract

**Background:**

*Cryptosporidium parvum* is a zoonotic parasitic protozoan that can infect a variety of animals and humans and is transmitted between hosts via oocysts. The oocyst wall provides strong protection against hostile environmental factors; however, research is limited concerning the oocyst wall at the proteomic level.

**Methods:**

A comprehensive analysis of the proteome of oocyst wall of *C. parvum* was performed using label-free qualitative high-performance liquid chromatography (HPLC) fractionation and mass spectrometry-based qualitative proteomics technologies. Among the identified proteins, a surface protein (CpSP1) encoded by the *C. parvum cgd7_5140* (*Cpcgd7_5140*) gene was predicted to be located on the surface of the oocyst wall. We preliminarily characterized the sequence and subcellular localization of CpSP1.

**Results:**

A total of 798 proteins were identified, accounting for about 20% of the CryptoDB proteome. By using bioinformatic analysis, functional annotation and subcellular localization of the identified proteins were examined for better understanding of the characteristics of the oocyst wall. To verify the localization of CpSP1, an indirect immunofluorescent antibody assay demonstrated that the protein was localized on the surface of the oocyst wall, illustrating the potential usage as a marker for *C. parvum* detection in vitro.

**Conclusion:**

The results provide a global framework about the proteomic composition of the *Cryptosporidium* oocyst wall, thereby providing a theoretical basis for further study of *Cryptosporidium* oocyst wall formation as well as the selection of targets for *Cryptosporidium* detection.

**Graphical Abstract:**

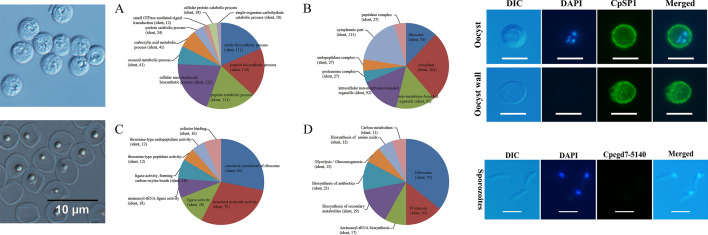

**Supplementary Information:**

The online version contains supplementary material available at 10.1186/s13071-022-05448-8.

## Background

*Cryptosporidium* is an intestinal parasitic protozoan capable of causing lethal diarrhea in immunocompromised people. In normal populations, the parasite infection results in self-limiting diarrhea. Among children under 5 years old, the prevalence of *Cryptosporidium* is second only to that of rotavirus [1–3]. At least 45 valid species and > 120 genotypes have been identified to date [4–6], of which *Cryptosporidium hominis* and *Cryptosporidium parvum* are widely transmitted among the human population, causing nearly 1 million deaths each year [7]. *Cryptosporidium* oocyst is extremely resistant to various physical and chemical interventions; for example, normal chlorine treatment is unable to remove the parasite from water, and this has caused several outbreaks in the past few decades [8]. To date, no effective drug or vaccine has been developed to fight this parasite. The only treatment approved by the US Food and Drug Administration (FDA) against *Cryptosporidiosis* is nitazoxanide, a drug that is effective for people with normal immune functions but ineffective for immunocompromised individuals [9, 10].

The oocyst stage of *C. parvum* plays an important role in its transmission. This stage in the life cycle is divided into thin-walled and thick-walled oocysts: thin-walled oocysts are used for self-infection, and thick-walled oocysts are excreted for the transmission of the parasite [8]. The composition of the oocyst wall is an important factor determining the survival of *C. parvum* in the external environment. The oocyst wall has been delineated by ultrastructural analysis; however, the specific protection mechanisms are not fully understood at the molecular level. Previous studies of the *C. parvum* oocyst wall has shown that it has a four-layered structure: a surface glycocalyx, a lipid hydrocarbon layer, a protein layer, and structural polysaccharides [11, 12]. The inner layer of cysteine-rich oocyst wall proteins (COWPs) forms disulfide bonds in the structure, preventing liquid intrusion and withstanding mechanical forces, thus being responsible for the rigidity of the oocyst wall [13]. In recent years, with the severe impact of several outbreaks of *C. parvum* in humans and domestic animals [14–16], interest in *C. parvum* oocysts has increased. Additionally, the genomes of *C. parvum* and *C. hominis* have been sequenced [17, 18]; with the rapid development of proteomics technology, the protein expression of *C. parvum* sporozoites during sporozoite excystation has been reported [19, 20]. The transcriptome during the development of *C. parvum* oocysts in vitro has been elucidated [21]. However, protein expression data are limited for the oocyst wall, which plays such a key role in the transmission and detection of this important zoonotic parasite.

In the present study, we isolated the *C. parvum* oocyst wall in vitro and analyzed its protein composition by label-free qualitative high-performance liquid chromatography (HPLC) fractionation and qualitative proteomics technologies. A total of 798 proteins were identified from 3978 unique peptides in oocyst walls, comprising approximately 20% of the whole predicted proteome of *C. parvum*. We also performed functional annotation and subcellular localization of the identified proteins. The analyses identified a protein that could be used for in vitro detection of *C. parvum*, a surface protein with two conserved cysteines (CpSP1) encoded by the *cgd7_5140* gene. We report the characterization of CpSP1 and its localization in the oocyst. Our overall goal was to identify the proteins expressed in the oocyst wall to better understand the role of the *Cryptosporidium* oocyst in the parasite’s life cycle. The results should provide a theoretical basis and new strategies for the mining of *Cryptosporidium* detection targets.

## Methods

### Oocyst preparation and oocyst wall purification

*Cryptosporidium parvum* oocysts of the IId strain (genotype A19G1 at the *gp60* locus) were maintained in the lab (International Joint Research Laboratory for Zoonotic Diseases of Henan) and continued to be passaged in newborn calves via collection of feces on the 3rd day after infection. The samples were purified as previously described [22]. For the purpose of the viability of the oocysts and the accuracy of the later experiments, the purified oocysts were stored at 4 °C for no more than 4 weeks. A total of 3 × 10^8^ oocysts were incubated with 2% sodium hypochlorite for 10 min at room temperature and then washed three times with PBS. Excystation was then performed at 37 °C by suspending in 0.75% taurocholic acid (sodium taurocholate, NaT) and 0.25% trypsin in PBS for 2 h [23, 24]. To obtain clean oocyst walls, 1 ml of the excysting solution was added to a high-speed centrifuge tube (Beckman Coulter, USA) containing 9 ml of 70% Percoll and centrifuged at 25,000 g for 45 min. The oocyst walls were aspirated with a pipette, washed three times with PBS and then centrifuged at 10,000 g for 10 min [22]. Micrographs of the purified oocyst wall were obtained using differential interference contrast microscopy. Images of 50 microscope fields per coverslip were captured randomly under 100 × magnification.

### Protein extraction and trypsin digestion

The protease inhibitor phenylmethylsulfonyl fluoride (PMSF) was added to the collected oocyst walls, after which the oocyst walls were frozen in liquid nitrogen for 3 min, thawed in a 37 °C water bath, and the steps repeated six times. PMSF and lysis buffer [3% sodium dodecyl sulfate (SDS), 8 M of urea] were added to break the oocyst walls, and the solution was lysed on ice for 30 min. The sample was sonicated at 100 W for 30 min and sonicated for 3 s with 8-s intervals, and the temperature and the occurrence of air bubbles were observed. The thermal cracking was followed by ultracentrifugation at 9000 × g for 30 min [25, 26]. Finally, the supernatant was collected, and the protein concentration was determined using a bicinchoninic acid (BCA) kit (Sangon, Shanghai, China) according to the manufacturer’s instructions. PMSF and 200 mM of dithiothreitol (DTT) were added, and the solution was stored at − 80 °C. For digestion, the protein solution was reduced with 5 mM of dithiothreitol for 30 min at 56 °C and alkylated with 11 mM of iodoacetamide for 15 min at room temperature in darkness. The protein sample was then diluted by adding 100 mM of tetraethylammonium bromide (TEAB) to urea at a concentration less than 2 M. Finally, trypsin was added at a 1:50 trypsin-to-protein mass ratio for the first digestion overnight and at a 1:100 trypsin-to-protein mass ratio for a second 4-h digestion.

### LC–MS/MS analysis of the *C. parvum* oocyst wall

LC–MS/MS and subsequent analysis were performed as described elsewhere [27]. The tryptic peptides were dissolved in 0.1% formic acid. The peptides were subjected to an NSI source, followed by tandem mass spectrometry (MS/MS) in a Q Exactive™ Plus (Thermo) coupled online to the UPLC. The peptide sequences were processed using the Maxquant search engine (v.1.5.2.8). Tandem mass spectra were searched against UniProt *Cryptosporidium_parvum* (6748 sequences) concatenated with a reverse decoy database. The cysteine alkylation was set as a fixed modification, and the variable modification was the oxidation of methionine, deamidation, and acetylation of the N-terminus of the protein. FDR for protein identification and PSM identification was set to 1%.

### Bioinformatic analysis

To thoroughly understand the functions of the identified proteins, we analyzed the characteristics of the proteins from the following aspects. Gene ontology (GO) annotations at the proteomics level were derived from the UniProt-GOA database (http://www.ebi.ac.uk/GOA/) [28]. These proteins were then classified according to cellular composition, molecular function, or physiological process. The protein domains were annotated using InterProScan and the corresponding InterPro domain database. The KEGG online service tool KAAS (http://www.genome.jp/kaas-bin/kaas_main) was used to annotate the submitted proteins; then, KEGG mapper (http://www.kegg.jp/kegg/mapper.html) was employed to match the annotated proteins to the corresponding pathways in the database [29]. We performed GO classification and KEGG pathway enrichment analysis on the identified proteins. The purpose was to determine whether the focal protein had a significant enrichment trend in certain functional types. Wolfpsort (http://www.genscript.com/psort/wolf_psort.html) and CELLO (http://cello.life.nctu.edu.tw/) were used to predict subcellular localization and for predictive analysis of submitted proteins.

### Production of recombinant proteins and preparation of polyclonal antibodies

Previous research has shown type I membrane proteins consist of a non-cytoplasmic domain that would be exposed to the extracellular side after trafficking to cytoplasmic membranes [30]. The full *cgd7_5140* gene was amplified from the genomic DNA of *C. parvum* II dA19G1 isolate using the primers 5’-CGCGGATCCATGAAGACTCATCTAGGATTGTCAGAG-3’ (the CGC protective base and BamHI restriction site underlined) and R:5’-CGCCTCGAGTTAGTTCATTAATTGATAAAAAAACTTACCGTCA-3’(the CGC protective base and XhoI restriction site underlined). The PCR was performed in a GeneAmp 2720 (Applied Biosystems) system with the following cycling conditions: 94 °C for 5 min; 35 cycles at 94 °C for 45 s, 60 °C for 45 s, 72 °C for 1 min, and 72 °C for 7 min and 4 °C storage. A Universal DNA Purification Kit (TIANGEN Biotech, Beijing, China) was used to obtain the recombinant product that was cloned into the pGEX-4 T-1 vector (Novagen, Madison, WI, USA). The recombinant plasmid was introduced into *Escherichia coli* DH5α cells, after which single colonies were chosen and used for PCR and sequencing to determine the accuracy.

The plasmid containing the correct sequence of the *cgd7_5140* gene was transformed into *E. coli* Rosetta (DE3) cells (TIANGEN Biotech, Beijing, China) for protein expression. Rosetta (DE3) cells were cultured in LB medium containing 100 μg/ml of ampicillin and grown at 37 °C until the OD_600_ reached 0.6–1.0, after which 0.1 mM of Isopropyl-beta-D-thiogalactopyranoside (IPTG) was added at 25 °C for 4 h to induce protein expression. SDS-PAGE and Western blot analyses were used to evaluate the expression level and solubility of the target protein.

To obtain the protein of interest, the bacteria were inoculated in 2 l medium and expressed as described above. The culture was centrifuged to collect bacteria and lysed by sonication on ice. The pellet was obtained by further centrifugation and resuspended with 0.5 mM of EDTA, 0.5 M of NaCl, and 20 mM of sodium phosphate and washed with PBS after centrifugation, after which 0.1% Triton-100 and 2 M, 4 M, and 6 M of urea were used to remove impurities. Finally, the pellet was resuspended with 8 M of urea, and the protein of interest was isolated using a PD-10 Desalting Column (GE Healthcare, Pittsburgh, PA, USA). A 10% SDS-PAGE gel was used for the identification of the protein.

Polyclonal antibodies against CpSP1 were generated in New Zealand white rabbits by Sangon Biotech (Shanghai, China). The priming antigen was mixed with an equal volume of Freund's complete adjuvant and emulsified; second, third, and fourth immune antigens were protein antigens mixed with an equal volume of Freund's incomplete adjuvant. On the 8th day after the fourth immunization, the anti-serum titer of the ELISA test met the requirements, and whole blood was collected from the arteries and then purified.

### Indirect immunofluorescence localization (IFA)

A small amount of *C. parvum* oocysts was placed into a 1.5-ml centrifuge tube and incubated with 1 ml of 0.5% sodium hypochlorite solution in an ice bath for 10 min. After washing three times with PBS, the oocysts were resuspended with PBS, after which 10 μl of the oocyst solution was placed on a glass slide and allowed to air dry. Then, methanol was added to fully cover the slide and fixed for about 30 min. The slide was then washed three times with PBS; 5% BSA solution was added, and the sample was incubated for 1 h and then washed three times with PBS. The anti-CpSP1 antibody was diluted with 5% BSA solution, incubated at 4 °C overnight, and washed three times with PBS. Next, AlexaFluor® 594-labeled goat anti-rabbit fluorescent secondary antibody (Bioss, Beijing, China) was diluted 1:200 fold with 5% BSA solution, and the oocysts were incubated for 1 h at room temperature in the dark and then washed three times with PBS. The cells were counterstained with the nuclear stain 4′, 6-diamidino-2-phenylindole (DAPI, Yeasen Biotech, Shanghai, China) and washed three times with PBS. A drop of Fluoromount-G™ (Yeasen Biotech) was added to set the cover slip, and the cover slips were fixed with nail polish. Fluorescence microscopy was performed using a BX53 immunofluorescence microscope (Olympus, Tokyo, Japan).

## Results

### Oocyst wall enrichment

The oocysts were successfully purified (Additional file 1: Fig. S1a), and most of the oocyst walls were recovered. Purified oocyst wall preparations contained virtually no sporozoites and were contaminated by approximately 0.8% intact oocysts. Most purified oocyst walls contained residual bodies (Additional file 1: Fig. S1b).

### Identification and description of proteins

Using LC–MS/MS with label-free qualitative HPLC, a total of 65,962 s-order spectra were obtained (comprising two technical replicates). After searching UniProt *Cryptosporidium_parvum*, the number of available valid spectra was 8555, and the percentage spectrum utilization was 13%. Then, through spectral analysis, a total of 4091 peptides were identified; the specificity was 3978, and 798 proteins were identified from the peptides (Additional file 2: Fig. S2a). The identified proteins comprised 612 previously described proteins and 186 uncharacterized proteins (Additional file 5: Table S1), and the top 20 most abundant proteins are listed in Table 1. The first-order mass error of most of the spectra was within 5 ppm, which accords with the high-precision characteristics of the orbital trap mass spectrometry (Additional file 2: Fig. S2b). The results indicate that the accuracy of the mass spectrometer was normal, and the qualitative analysis of the proteins should not be affected by large mass deviations.

**Table 1:** List of the top 20 abundant proteins in this study

Gene name	Description	MW (kDa)	Score	Previously identified? (Yes/No)	Location
1 MB.208	Oocyst EB module wall protein	175.79	323.31	No	NA
COWP3	Oocyst wall protein 3	106.43	323.31	Yes	Oocyst wall
COWP6	Oocyst wall protein 6	58.616	323.31	Yes	Oocyst wall
COWP8	Oocyst wall protein 8	49.99	323.31	Yes	Oocyst wall
cgd7_300	CpCCP2	184.53	323.31	No	NA
cgd7_1730	CpCCp1/Cpa135	172.76	323.31	Yes	The apical pole of sporozoites
cgd8_2790	Pleckstrin homology (PH domain) containing protein	475.45	323.31	No	NA
cgd3_3370	Uncharacterized protein	165.85	323.31	No	NA
cgd2_790	CpCCp3	134.53	323.31	No	NA
cgd4_3530	Uncharacterized protein	175.38	323.31	No	NA
HSP70	Heat shock protein 70	70.189	323.31	No	NA
cgd7_3670	Heat shock protein 90 (Hsp90), signal peptide plus ER retention motif	89.192	306.94	No	NA
COWP7	Oocyst wall protein 7	93.94	299.23	Yes	Oocyst wall
1MB.267	Alpha-1,4 glucan phosphorylase	104.21	273.51	No	NA
cgd6_120	Protein disulfide isomerase, probable	53.788	270.63	No	NA
cgd6_1080	60 kDa glycoprotein	28.64	270.35	Yes	Sporozoites, parasitophorous vacuole membrane (PVM)
cgd8_1720	Acetaldehyde reductase plus alcohol dehydrogenase (AdhE) of possible bacterial origin (fragment)	94.734	251.12	No	NA
cgd6_4460	Large protein with ARM repeats (fragment)	278.39	222.55	No	NA
cgd7_320	60S ribosomal protein L18a	21.465	216.49	No	NA
cgd7_1340	Uncharacterized protein	123.73	215.87	No	NA

**Fig. 1:**
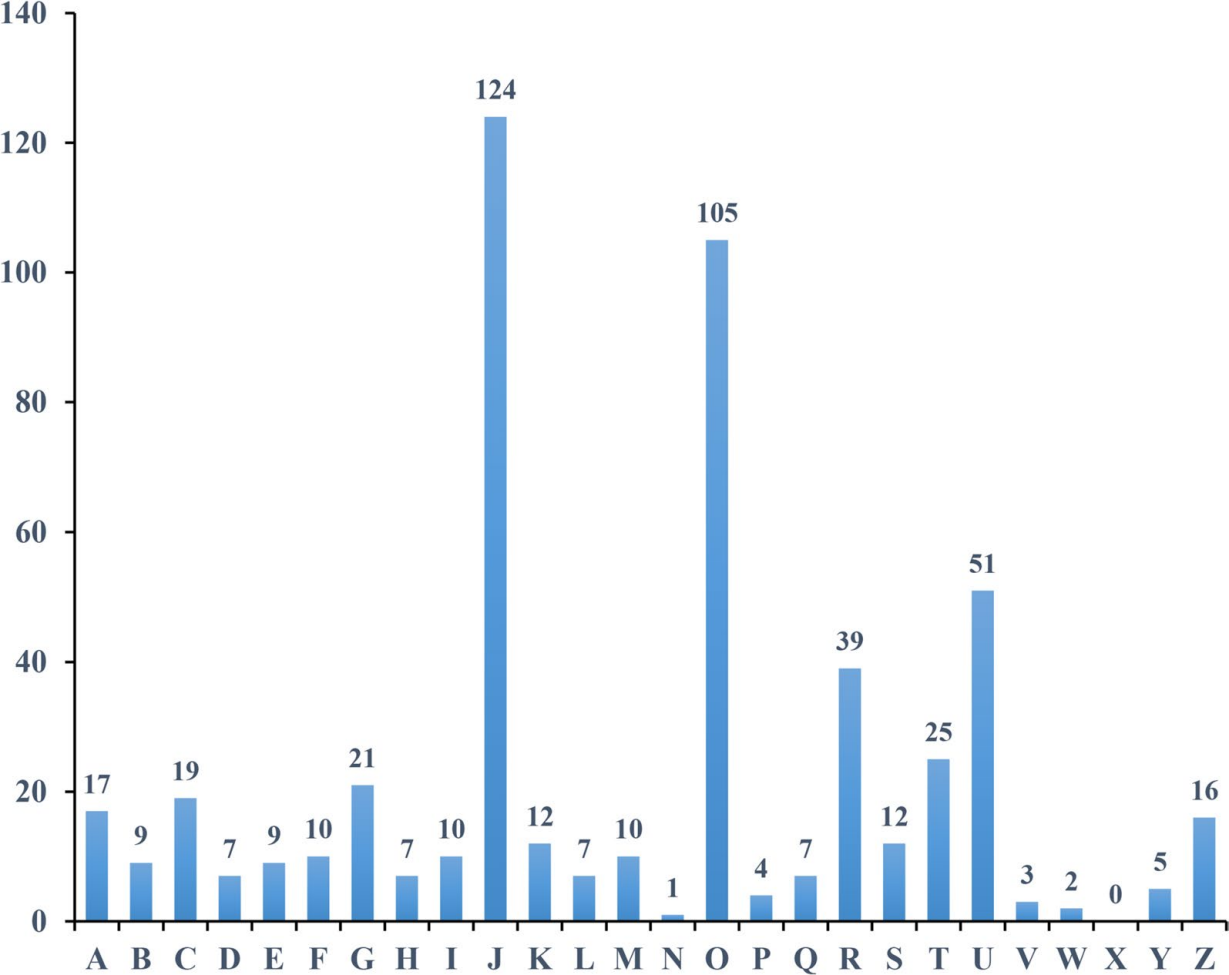
Cluster of Orthologous Groups (COG) analysis of the identified proteins in *C. parvum* oocyst walls. The y-axis represents the number of proteins. The x-axis represents different COG classes. **A** RNA processing and modification; **B** chromatin structure and dynamics; **C** energy production and conversion; **D** cell cycle control, cell division, chromosome partitioning; **E** amino acid transport and metabolism; **F** nucleotide transport and metabolism; **G** carbohydrate transport and metabolism; **H** coenzyme transport and metabolism; **I** lipid transport and metabolism; **J** translation, ribosomal structure, and biogenesis; **K** transcription; **L** replication, recombination, and repair; **M** cell wall/membrane/envelope biogenesis; **N** cell motility; **O** posttranslational modification, protein turnover, chaperones; **P** inorganic ion transport and metabolism; **Q** secondary metabolites biosynthesis, transport, and catabolism; **R** general function prediction only; **S** function unknown; **T** signal transduction mechanisms; **U** intracellular trafficking, secretion, and vesicular transport; **V** defense mechanisms; **W** extracellular structures; **X** mobilome: prophages, transposons; **Y** nuclear structure; **Z** cytoskeleton

For the 798 proteins identified, we performed subcellular localization by the general subcellular predictors Wolfpsort and CELLO (Additional file 3: Fig. S3). Proteins with cytoplasm (34.9%) localizations were highly represented. The localizations’ extracellular (18.7%) and plasma membrane (13.7%) associated with the parasite's secretory pathway accounted for a third of the predicted molecules. Most of the proteins had been characterized in previous studies. For the Clusters of Orthologous Groups (COG) analysis of proteins, approximately 66.7% were covered and classified into 25 categories (Fig. 1). As can be seen from the figure, the three categories with the most identified proteins were translation, ribosomal structure, and biogenesis; posttranslational modification, protein turnover, and chaperones; and intracellular trafficking, secretion, and vesicular transport.

### Functional annotation of proteins

**Fig. 2:**
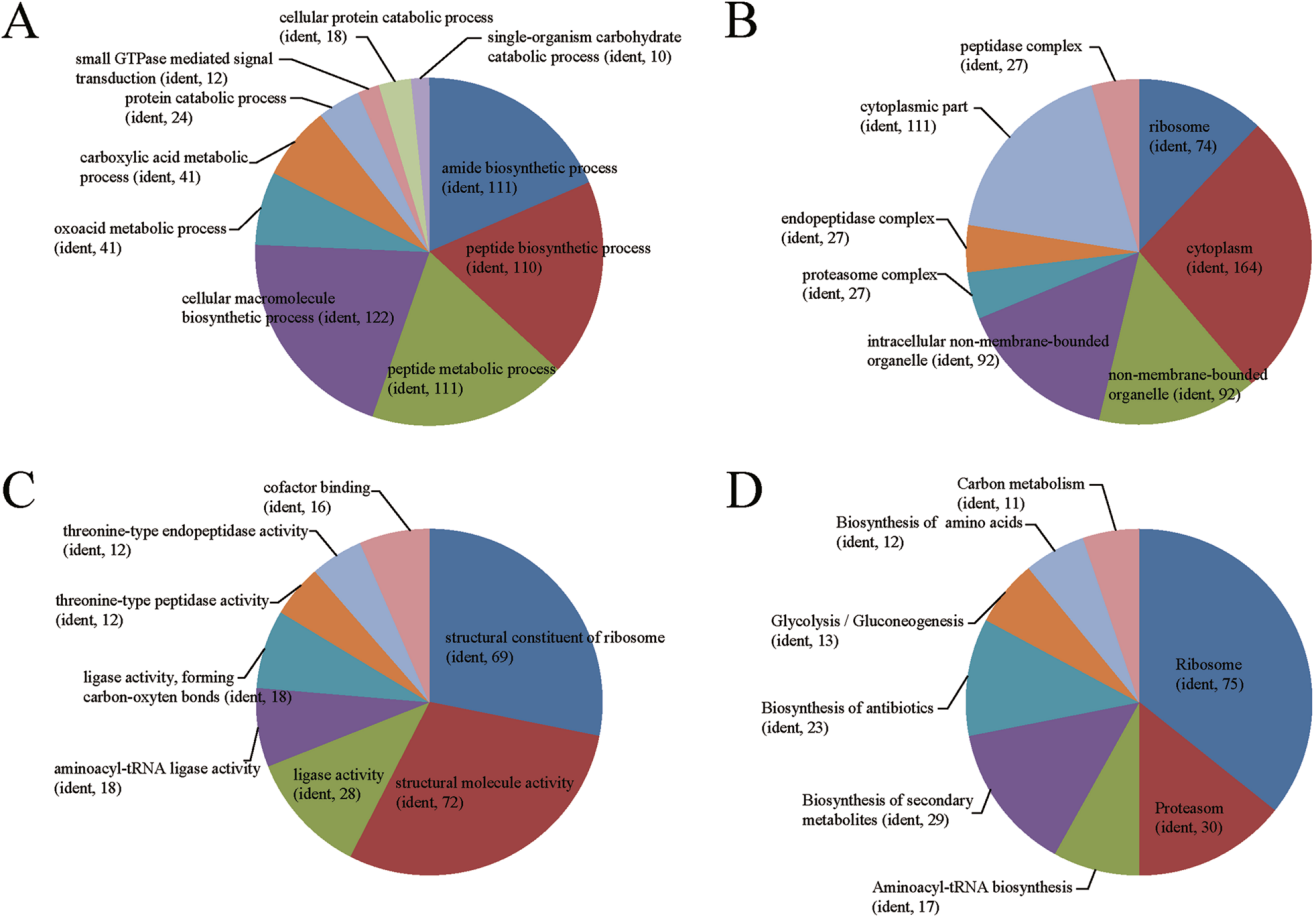
Gene ontology (GO) and KEGG pathway distribution of identified proteins in *C. parvum* oocyst walls. **A** Top ten enriched GO terms under “biological process”; **B** Top eight enriched GO terms under “cellular components.” **C** Top eight enriched GO terms under “molecular function.” **D** Top eight enriched KEGG pathways. ident, number of identified proteins

To further elucidate the functions of the identified proteins, combined with the UniProt-GOA data (http://www.ebi.ac.uk/GOA/) used to annotate the proteins, the top eight enriched GO terms within the three major categories (biological processes, molecular functions, and cell composition) were identified in protein enrichment analysis (Fig. 2). The top two most enriched GO terms under molecular function were structural molecule activity and structural constituent of ribosome. In the biological process category, cellular macromolecule biosynthetic process, peptide metabolic process, amide biosynthetic process, and peptide biosynthetic process were prominent categories. The top four enriched GO terms under cellular component were ribosome, cytoplasmic part, non-membrane-bounded organelle, and intracellular non-membrane-bounded organelle. Interestingly, the ribosome term included 164 related proteins, making it the most enriched GO term among other sub-categories under the three major functional categories.

Through KEGG pathway enrichment analysis, among these 798 identified proteins, only 268 had a KEGG Orthology (KO) ID, and these were related to 11 pathways. The eight significant enriched pathways were ribosome, proteasome, aminoacyl-tRNA biosynthesis, biosynthesis of secondary metabolites, biosynthesis of antibiotics, glycolysis/gluconeogenesis, biosynthesis of amino acids, and carbon metabolism (Fig. 2D).

### Expression of recombinant CpSP1

**Fig. 3:**
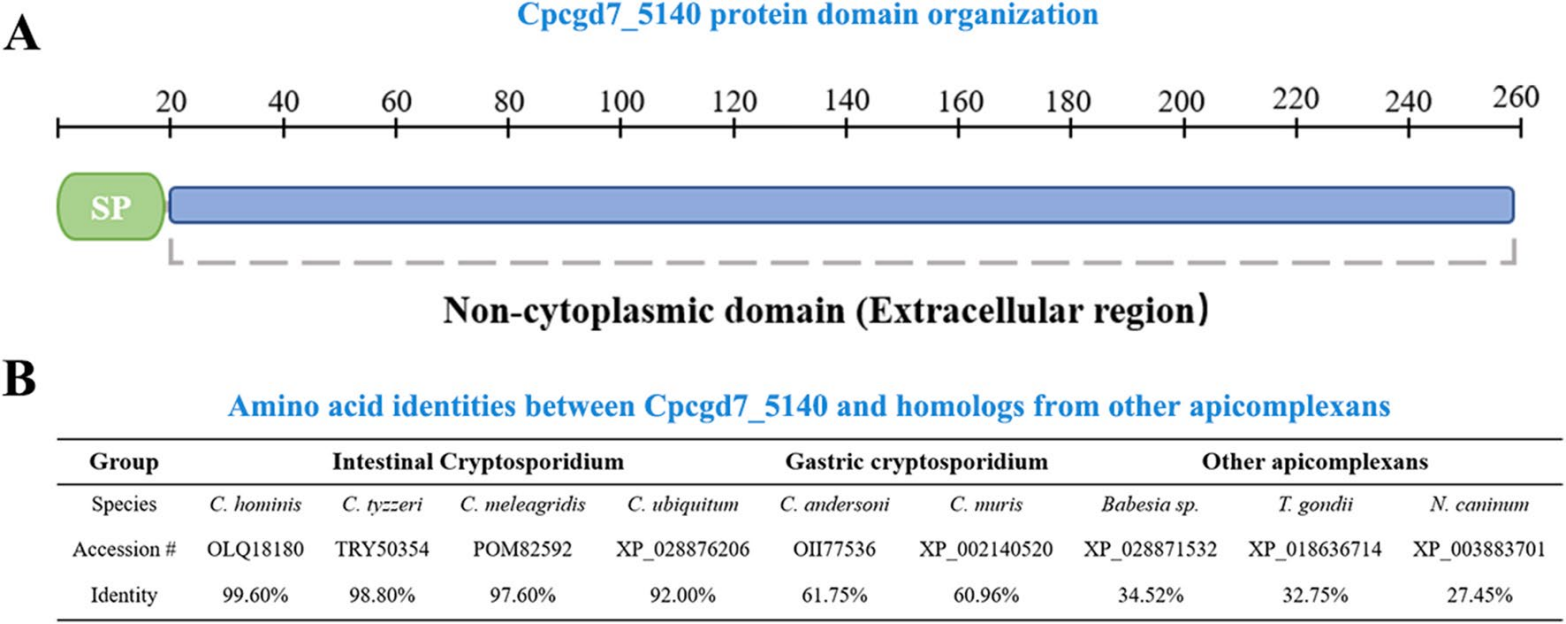
*Cryptosporidium parvum* CpSP1 sequence organization **A** and amino acid identities with orthologs from other *Cryptosporidium* species and other apicomplexans **B**. SP: signal peptide. Full names for list of other apicomplexans: *Babesia* spp., *Toxoplasma gondii*, *Neospora caninum*

**Fig. 4:**
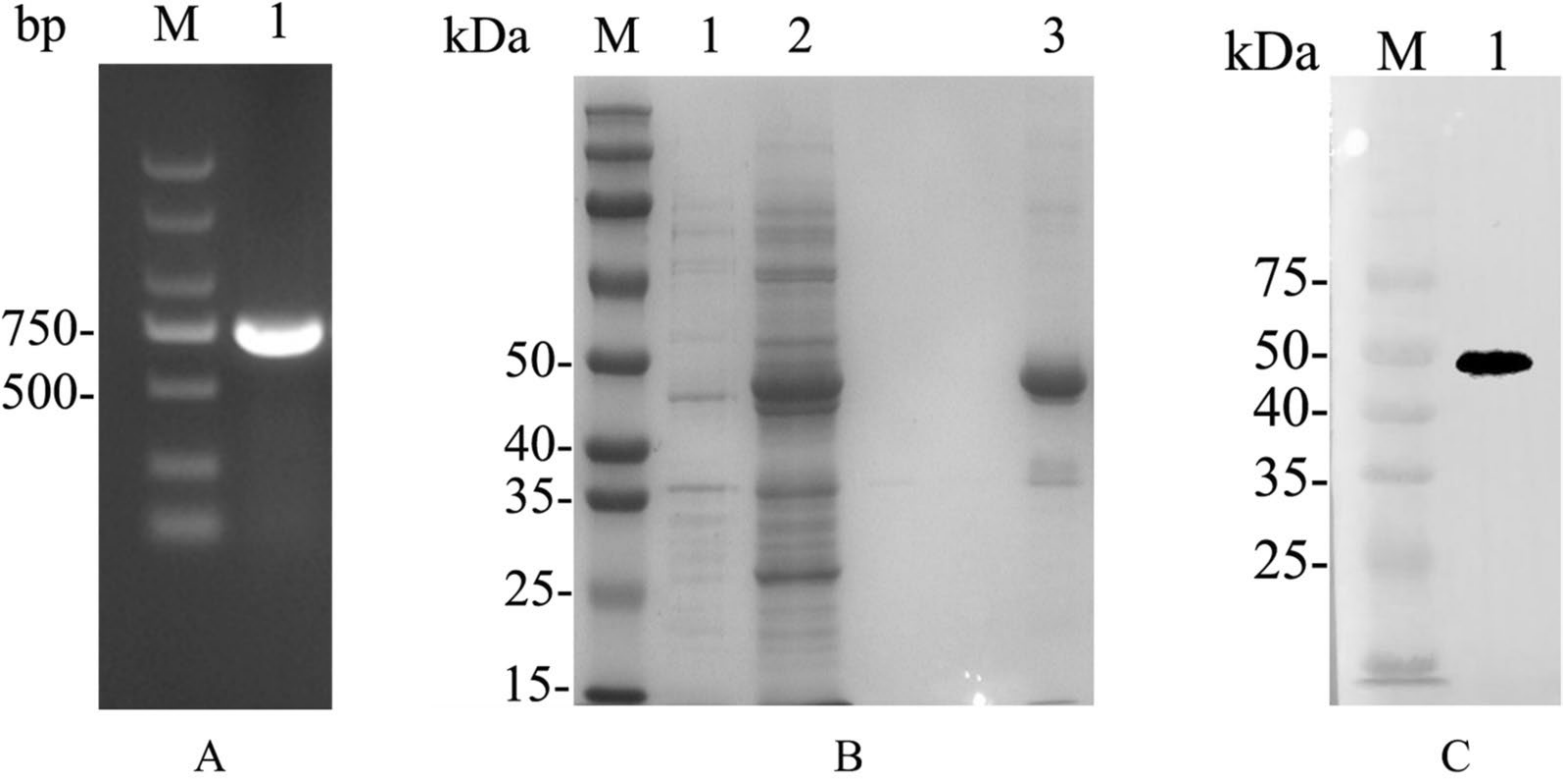
Expression of CpSP1 in *Escherichia coli*. **A** PCR amplification of the *Cpcgd7_5140* gene of *C. parvum*. Lane M: 2000-bp DNA ladder marker. Lane 1: *Cpcgd7_5140* product. **B** Analysis of CpSP1 expressed in *E. coli* and purity of recombinant CpSP1 revealed by SDS-PAGE. Lane 1: lysate from bacterial cells transformed with pGEX-4t-1-*Cpcgd7_5140* without IPTG induction. Lane 2: lysate from bacterial cells transformed with pGEX-4t-1-*Cpcgd7_5140* with IPTG induction. Lane 3: CpSP1 purified using gradient urea and PD-10 desalting columns. **C** Western blot analysis of recombinant CpSP1 in *E. coli* using Anti-GST tag. Lane M: protein size markers. Lane 1: lysate from bacterial cells transformed with pGEX-4t-1-*Cpcgd7_5140* with IPTG induction

Through bioinformatic analyses, we identified a protein that may be located on the surface of the oocyst wall: a surface protein with two conserved cysteines (CpSP1), containing a non-cytoplasmic domain (Fig. 3A). The protein was highly conserved only within the *Cryptosporidium* species, especially within the intestinal *Cryptosporidium* species (Fig. 3B). The *Cpcgd7_5140* gene amplified from genomic DNA was extracted from *C. parvum* II d strain (Fig. 4A) and cloned into the pMD-18 T vector. The protein was then expressed in *E. coli* Rosetta (DE3) cells (Fig. 4B). In SDS-PAGE analysis of the recombinant CpSP1, the expected molecular mass with a predicted size of ~ 48 kDa was observed. The recombinant CpSP1 was also confirmed by Western blot analysis using anti-GST tag antibodies (Fig. 4C). Finally, the recombinant CpSP1 was purified with gradient urea, and the target protein was eluted by using PD-10 desalting columns.

### CpSP1 is located in *C. parvum* oocyst wall

**Fig. 5:**
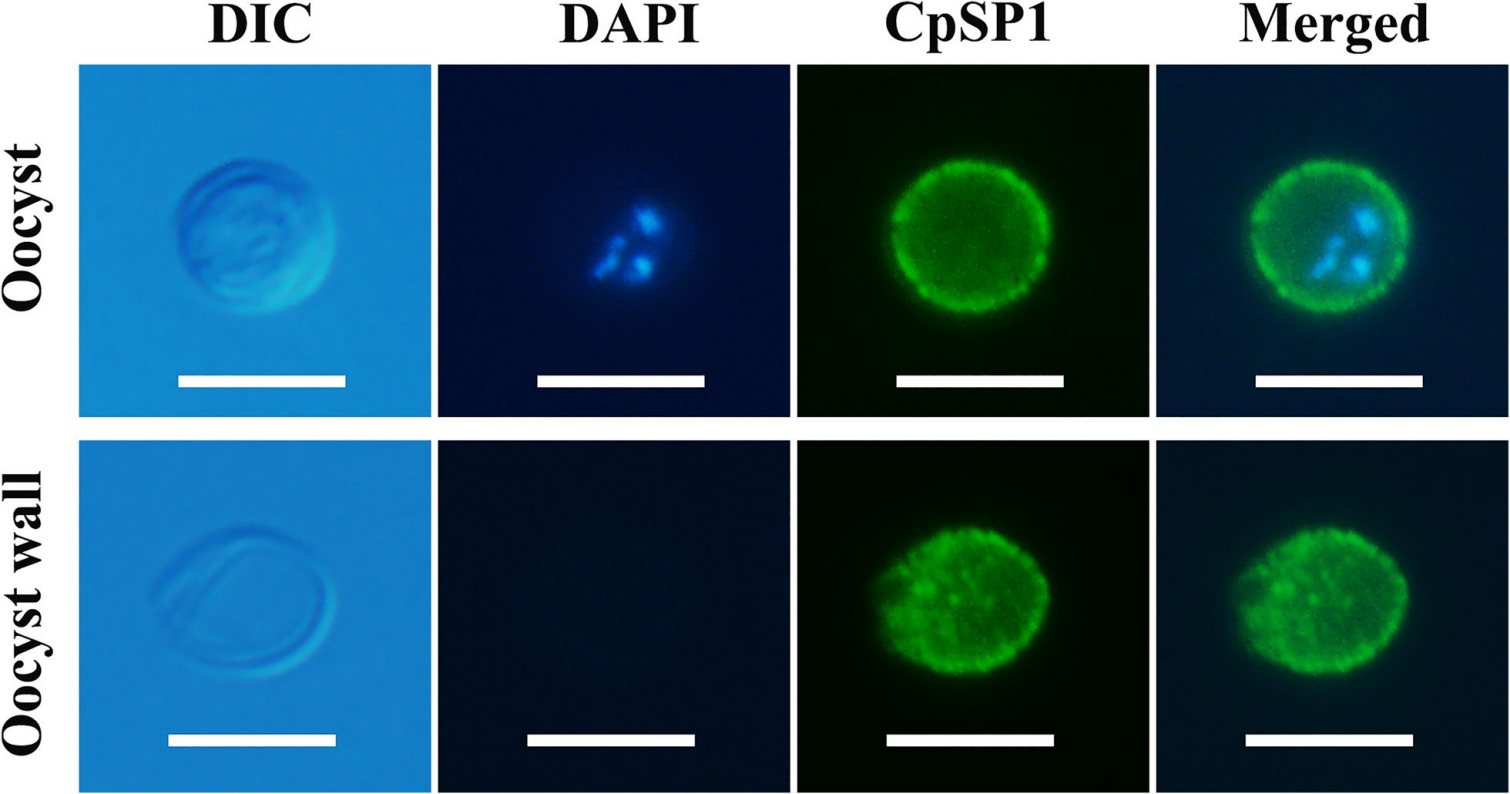
Immunofluorescence microscopy detection of CpSP1 in *C. parvum* oocyst stage using the rabbit anti-CpSP1 antibody. Scale bars: 5 μm

The localization of CpSP1 in the *C. parvum* oocyst was examined by immunofluorescence microscopy. When observed with an immunofluorescence microscope, the anti-CpSP1 antibody was seen to be located on the entire free oocyst (Fig. 5). No signals were observed in parasites when using pre-immune sera. In addition, no significant signals were observed in the free sporozoite stage when using anti-CpSP1 polyclonal antibody (Additional file 4: Fig. S4).

## Discussion

*Cryptosporidium* is a diarrhea-causing pathogen, and its medical and veterinary importance are second only to those of rotavirus [3, 31]. The *C. parvum* oocyst is a major stage in the processes of parasite transmission and infection. The oocyst wall plays a key role in maintaining the oocysts in the natural environment during this stage [11, 32]. Therefore, understanding the composition and proteins of the *Cryptosporidium* oocyst wall is important for understanding the ability of *Cryptosporidium* to cope with harsh environments and for the development of new detection targets. In this study, we first reported the identification of 798 proteins expressed in the thick-walled *C. parvum* oocyst wall, an important structure that has rarely been described at the molecular level despite its decisive role in parasite transmission. The examined material was composed of the thick-walled oocyst wall and residual body after excystation. The identified proteins represented roughly 20% of the predicted proteome [17]. While the presence of residual body in the sample may give a small gap between the composition of oocyst wall-associated proteins identified in this study and the true composition of oocyst wall proteins, it provides more extensive protein data, including abundantly expressed protein inside the *C. parvum* oocyst walls after excystation.

Through enriched GO terms and enriched KEGG pathways, a large number of ribosomal and proteasome-related proteins were identified, indicating the importance of ribosomal proteins and proteasome after decapsulation. Previous studies have shown that most of these proteins are involved in energy metabolism, protein synthesis, and excystation [8, 19]. The identification of ribosomal and proteasome-related proteins has been observed in other apicomplexan protozoan parasites (e.g. *Toxoplasma gondii*) [33, 34]. Regarding the strong expression of energy metabolism and ribosomal-related proteins, this was probably due to the need to quickly activate a series of protein synthesis steps after excystation [19, 20]. Proteasome-related proteins play a significant role in the differentiation and invasion stages of parasites, and they can also be used as virulence factors [35]. Blocking of proteasome function can prevent parasite growth and reproduction, as has been confirmed in both *Cryptosporidium* and *Toxoplasma* [36, 37]. This provides new strategies for the development of anti-parasite drugs. In our samples, we identified proteins that were localized on sporozoites or secreted by sporozoites to promote gliding motility. For example, three proteins containing LCCL domains (Cpa135, CpCC2, and CpCC3) were detected, and previous studies have suggested that these proteins are involved in the gliding and adhesion processes [19, 38]. We also identified a number of surface and extracellular proteins, suggesting contamination in the oocyst wall sample or that these proteins are perhaps expressed on the oocyst wall or residual sporozoite body.

A series of classical oocyst wall proteins (COWP1-9) were identified in the extract of *C. parvum* oocyst wall, and they had high abundance, which proved that the oocyst wall extracted in this study had reliable purity. The proteins are cysteine-rich peptides expressed during oocyst formation that maintain oocyst toughness by forming disulfide bonds; the proteins thus could also function in the oocyst wall as structural proteins [39, 40]. Previous studies have shown that COWP1-9 are localized to the inner oocyst wall and thus are not as appropriate for sample detection in the environment [41]. Based on the idea that structurally similar components may contribute to the same functions, the *Cpcgd7_5140* gene encodes a surface protein with two conserved cysteines, possibly through the formation of disulfide bonds in the structure of the oocyst wall and thus could be employed as an in vitro detection target. In this study, we identified, expressed, and characterized this protein and found that the recombinant CpSP1 was mainly expressed in inclusion bodies in the *E. coli* lysate. We located this protein on the oocyst by IFA, demonstrating that the protein encoded by the *Cpcgd7_5140* gene may be a candidate for the detection of cryptosporidiosis.

## Conclusion

Our study is the first using a label-free qualitative-based proteomic approach to analyze the proteins expressed in the oocyst walls collected after the excystation of *C. parvum*, thereby furthering our understanding of the molecular composition of the oocyst wall and improving the protein expression profile. It is worth noting that several cysteine-containing proteins may be new candidates for *Cryptosporidium* detection. Hopefully, subsequent research on these types of candidates will enable the rapid detection of *C. parvum* oocysts in the environment and reduce the spread of this parasite between humans and animals in future.

## Supplementary Information

**Additional file 1: Figure S1**. Purified *C. parvum* oocysts (a) and oocyst walls (b) under differential interference contrast microscopy. Scale bars: 10 μm.

**Additional file 2: Figure S2.** Label-free qualitative analysis coupled with LC-MS/MS analysis of *C. parvum* oocyst walls. (a) Basic statistical results of oocyst wall samples analyzed by mass spectrometry. The y-axis represents the number of proteins. (b) Mass accuracy distribution of mass spectrometry. The x-axis represents the first-order mass error range of the spectrum. The y-axis represents the score of spectrum matching peptides (characteristics of characterization of peptide identification).

**Additional file 3: Figure S3**. Subcellular localization chart of identified proteins in *C. parvum* oocyst walls.

**Additional file 4: Figure S4.** Immunofluorescence microscopy detection of CpSP1 in *C. parvum*-free sporozoites stage using the rabbit anti-CpSP1 antibody. Scale bars: 5 μm.

**Additional file 5: Table S1**. Full proteins' information detected from the *C. parvum* oocyst walls.

## Data Availability

The mass spectrometry proteomics data have been deposited to the ProteomeXchange Consortium via the PRIDE [42] partner repository with the dataset identifier PXD029431.

## Abbreviations

HPLC: High-performance liquid chromatography
IFA: Immunofluorescent antibody assay
FDA: Food and Drug Administration
COWPs: Oocyst wall proteins
GO: Gene ontology
KEGG: Kyoto Encyclopedia of Genes and Genomes
COG: Clusters of orthologous groups
LCCL: Limulus factor C cochlear protein Coch-5b2 lung
