# Supplementary figures and images for: Enrichment and proteomic identification of Cryptosporidium parvum oocyst wall
Source: Parasit Vectors. 2022 Sep 23;15:335. doi: 10.1186/s13071-022-05448-8 (PMC9508764; doi:10.1186/s13071-022-05448-8)

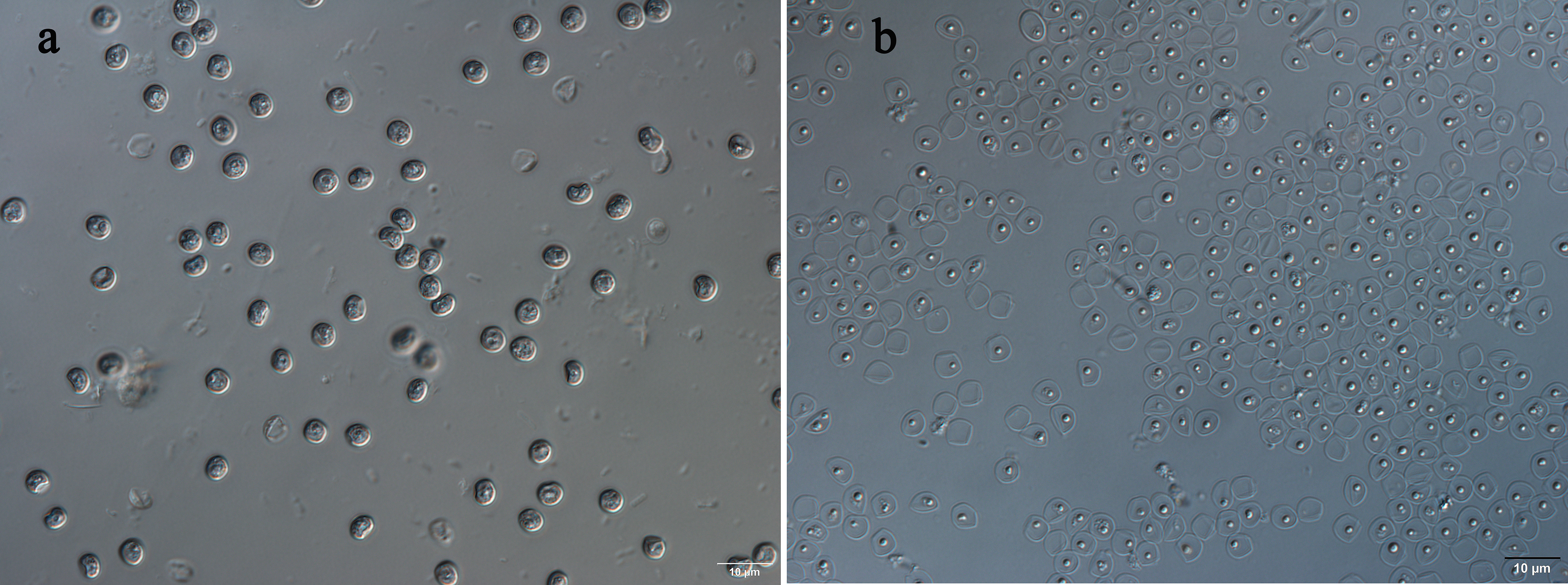

Supplement: Supplementary file 1 — Additional file 1: Figure S1. Purified C. parvum oocysts (a) and oocyst walls (b) under differential interference contrast microscopy. Scale bars: 10 μm. [file 13071_2022_5448_MOESM1_ESM.tif]

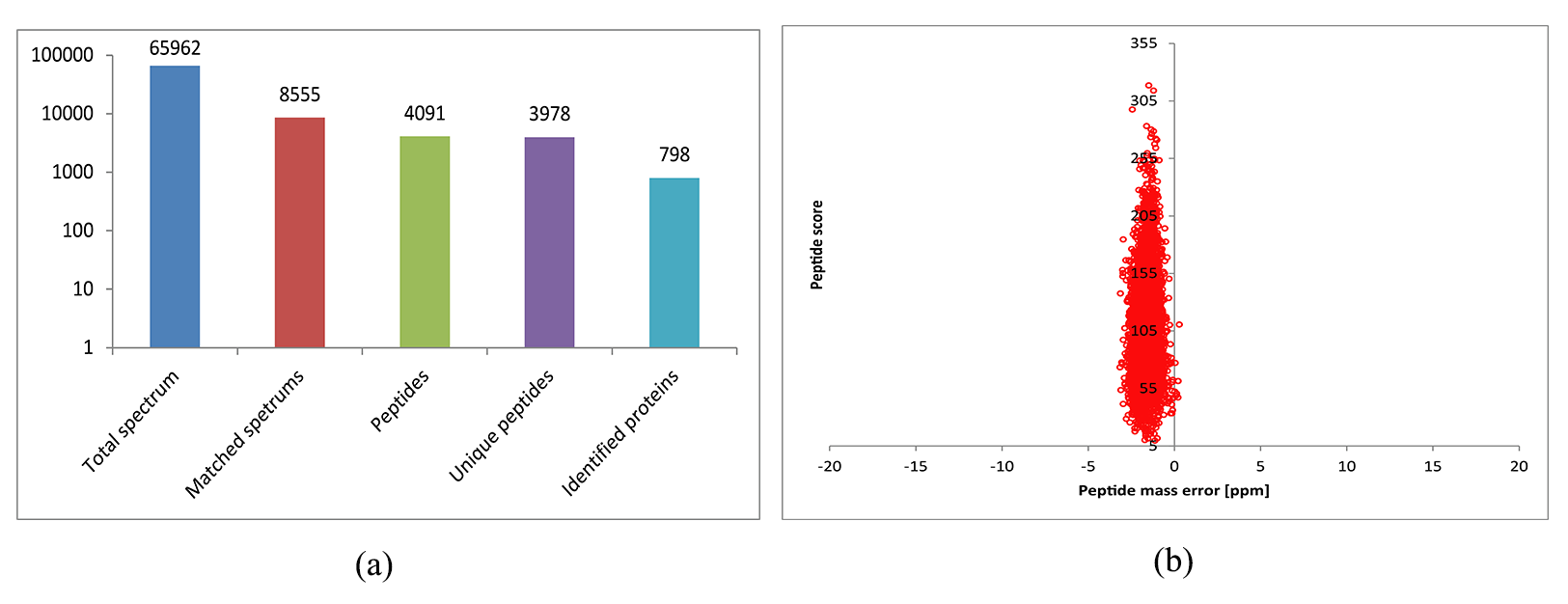

Supplement: Supplementary file 2 — Additional file 2: Figure S2. Label-free qualitative analysis coupled with LC-MS/MS analysis of C. parvum oocyst walls. (a) Basic statistical results of oocyst wall samples analyzed by mass spectrometry. The y-axis represents the number of proteins. (b) Mass accuracy distribution of mass spectrometry. The x-axis represents the first-order mass error range of the spectrum. The y-axis represents the score of spectrum matching peptides (characteristics of characterization of peptide identification). [file 13071_2022_5448_MOESM2_ESM.tif]

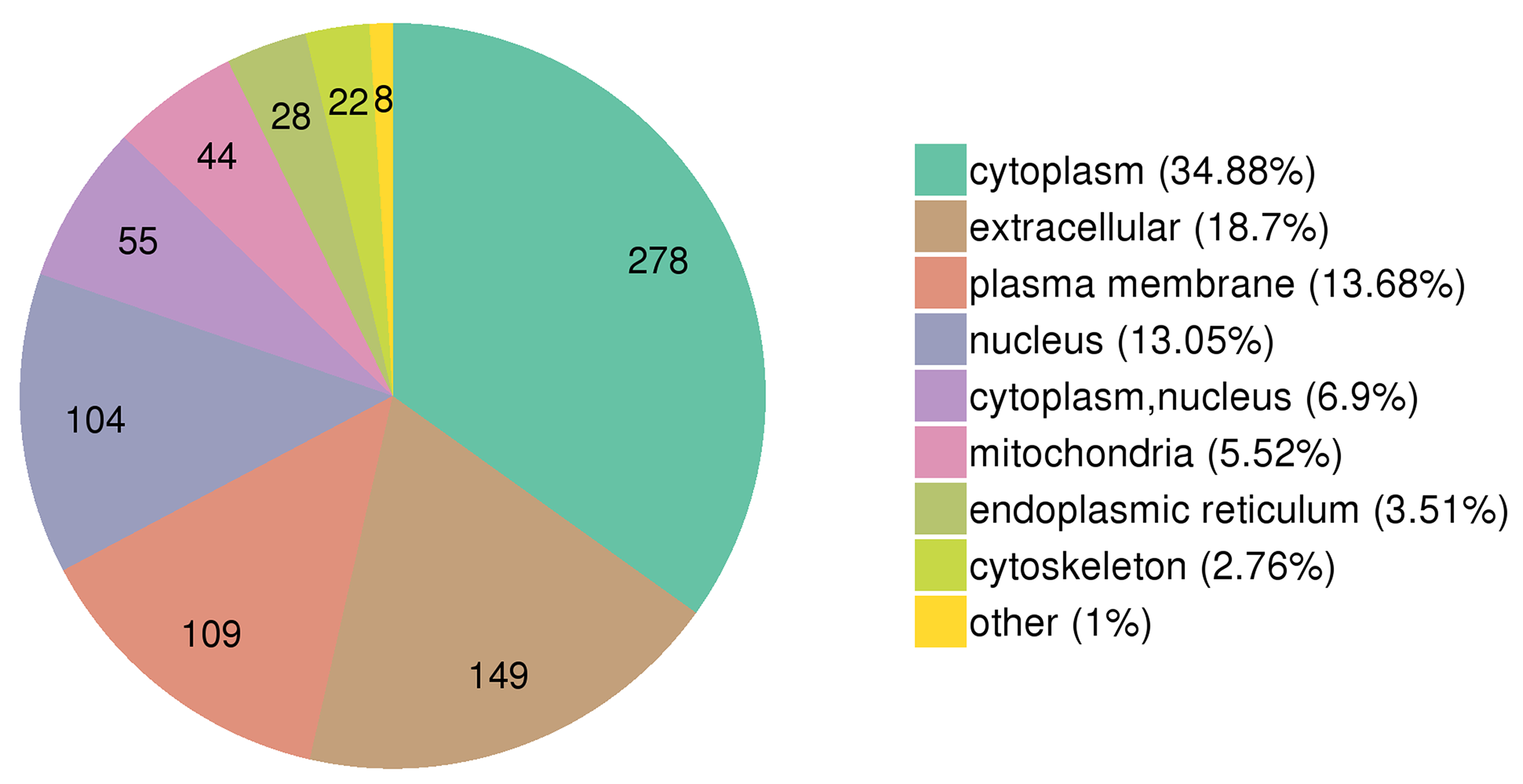

Supplement: Supplementary file 3 — Additional file 3: Figure S3. Subcellular localization chart of identified proteins in C. parvum oocyst walls. [file 13071_2022_5448_MOESM3_ESM.tif]

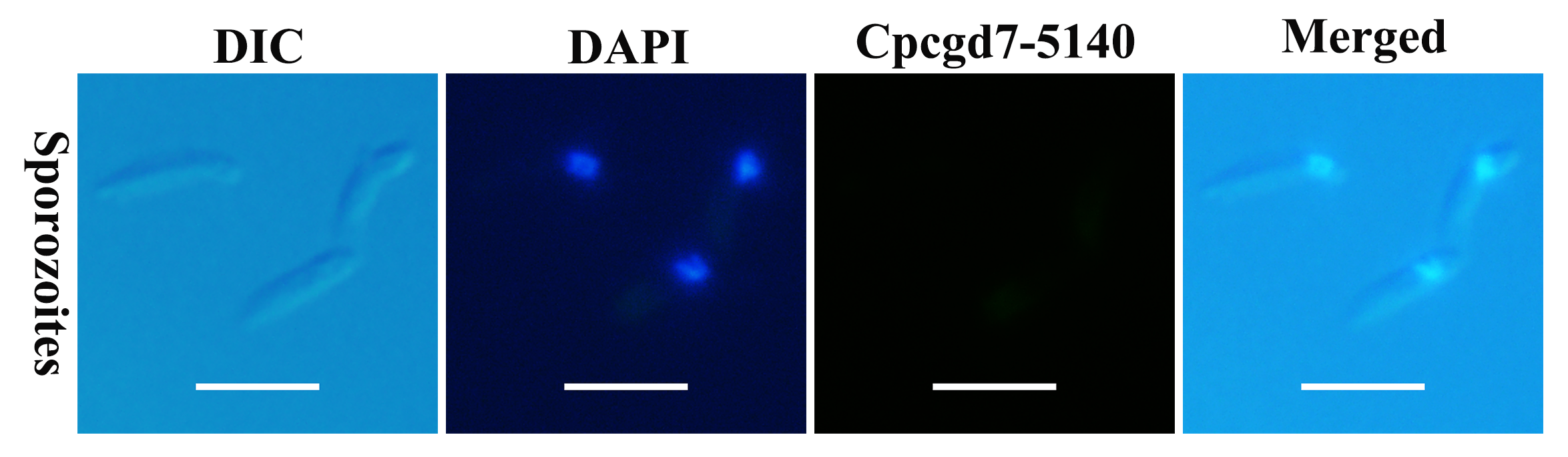

Supplement: Supplementary file 4 — Additional file 4: Figure S4. Immunofluorescence microscopy detection of CpSP1 in C. parvum-free sporozoites stage using the rabbit anti-CpSP1 antibody. Scale bars: 5 μm. [file 13071_2022_5448_MOESM4_ESM.tif]
