# Supplementary material for: Enrichment and proteomic identification of Cryptosporidium parvum oocyst wall
Source: Parasit Vectors. 2022 Sep 23;15:335. doi: 10.1186/s13071-022-05448-8 (PMC9508764; doi:10.1186/s13071-022-05448-8)
Supplement: Supplementary file 5 — Additional file 5: Table S1. Full proteins' information detected from the C. parvum oocyst walls. [file 13071_2022_5448_MOESM5_ESM.docx]

**Table S1 Full Proteins information detected from the *Cryptosporidium* *parvum* oocyst walls**

| **Protein accession** | **Protein description** | **Gene name** | **MW (kDa)** | **Score** | **Coverage (%)** | **Peptides** | | **PSMs** | **Unique peptides** |
| --- | --- | --- | --- | --- | --- | --- | --- | --- | --- |
| Q7YYI4 | Oocyst EB module wall protein | 1MB.208 | 175.79 | 323.31 | 61.8 | 80 | 743 | | 2 |
| Q6S5J1 | Oocyst wall protein 3 | COWP3 | 106.43 | 323.31 | 43 | 36 | 78 | | 36 |
| Q6S5I8 | Oocyst wall protein 6 | COWP6 | 58.616 | 323.31 | 61.9 | 25 | 124 | | 25 |
| Q6S5I7 | Oocyst wall protein 8 | COWP8 | 49.99 | 323.31 | 57.3 | 19 | 301 | | 10 |
| Q5CZ08 | CpCCP2 extracellular protein with a signal peptide, ricin, discoidin, NEC, LCCL, 2 levanase and an apicomplexan-specific cysteine-rich repeat (ApicA repeat) | cgd7_300 | 184.53 | 323.31 | 33.6 | 46 | 88 | | 46 |
| Q5CYM9 | CpCCp1/Cpa135 | cgd7_1730 | 172.76 | 323.31 | 29.4 | 39 | 61 | | 39 |
| Q5CVU0 | Pleckstrin homology (PH domain) containing protein | cgd8_2790 | 475.45 | 323.31 | 20.3 | 75 | 95 | | 75 |
| Q5CUC6 | Uncharacterized protein | cgd3_3370 | 165.85 | 323.31 | 37.4 | 48 | 103 | | 48 |
| Q5CU06 | CpCCp3, multidomain extracellular protein with a signal peptide and the following architecture: LH2+LCCL+2xSR+LCCL+pentraxin+2xLCCL | cgd2_790 | 134.53 | 323.31 | 33.1 | 34 | 56 | | 34 |
| Q5CQS3 | Uncharacterized protein | cgd4_3530 | 175.38 | 323.31 | 35.9 | 49 | 73 | | 49 |
| A6XNE8 | Heat shock protein 70 (Fragment) | HSP70 | 70.189 | 323.31 | 49.5 | 32 | 63 | | 31 |
| Q5CY50 | Heat shock protein 90 (Hsp90), signal peptide plus ER retention motif | cgd7_3670 | 89.192 | 306.94 | 40.9 | 29 | 48 | | 29 |
| Q6S5I5 | Oocyst wall protein 7 | COWP7 | 93.94 | 299.23 | 27.6 | 20 | 50 | | 20 |
| Q7YYF0 | Alpha-1,4 glucan phosphorylase | 1MB.267 | 104.21 | 273.51 | 35.3 | 33 | 41 | | 33 |
| Q7YY73 | Protein disulphide isomerase, probable | 56k.11 | 53.788 | 270.63 | 49.9 | 21 | 46 | | 21 |
| A0A1Z3FUV3 | 60 kDa glycoprotein (Fragment) | --- | 28.64 | 270.35 | 50.5 | 13 | 47 | | 0 |
| Q5CW41 | Acetaldehyde reductase plus alcohol dehydrogenase (AdhE) of possible bacterial origin (Fragment) | cgd8_1720 | 94.734 | 251.12 | 32.2 | 24 | 34 | | 24 |
| Q5CWM7 | Large protein with ARM repeats (Fragment) | cgd6_4460 | 278.39 | 222.55 | 19.7 | 45 | 61 | | 45 |
| Q5CZ06 | 60S ribosomal protein L18a | cgd7_320 | 21.465 | 216.49 | 53.5 | 12 | 36 | | 12 |
| Q5CYR2 | Uncharacterized protein | cgd7_1340 | 123.73 | 215.87 | 26.9 | 28 | 44 | | 28 |
| Q5CT01 | Signal peptide, large protein | cgd1_640 | 147.43 | 213.53 | 32.5 | 38 | 44 | | 38 |
| Q5CXZ8 | Signal peptide, large secreted protein | cgd7_4280 | 120.69 | 212.17 | 31.4 | 31 | 52 | | 31 |
| Q5CZ02 | Heat shock protein, Hsp70 | cgd7_360 | 71.87 | 211.32 | 49.6 | 32 | 53 | | 31 |
| Q7JQE2 | Elongation factor 1-alpha | cgd6_3990 | 48.161 | 209.4 | 60.9 | 24 | 54 | | 24 |
| Q7YYQ9 | Glyceraldehyde-3-phosphate dehydrogenase | cgd6_3790 | 36.125 | 208.56 | 64.6 | 17 | 37 | | 17 |
| Q5CXV2 | CpFNPA, extracellular protein with a signal peptide, FN2 domain and an anthrax toxin-N-terminus like domain. starts at second Met with signal peptide following | cgd7_4810 | 80.006 | 201.59 | 38.9 | 27 | 45 | | 27 |
| Q5CYA3 | Pyruvate decarboxylase (Fragment) | cgd7_3120 | 65.728 | 194.95 | 35.5 | 20 | 49 | | 20 |
| D7US15 | Thrombospondin-related adhesive protein (Fragment) | TRAP-C1 | 59.575 | 193.91 | 35.3 | 18 | 31 | | 16 |
| Q6S5J2 | Oocyst wall protein 2 | COWP2 | 153.67 | 191.39 | 33.5 | 40 | 62 | | 40 |
| F0X3V0 | Cgd2_810 protein | cgd2_810 | 16.764 | 186.39 | 70.5 | 11 | 22 | | 11 |
| F0X4N6 | Cgd8_430 protein | cgd8_430 | 29.348 | 181.5 | 38.6 | 12 | 22 | | 12 |
| F0X5K7 | Cgd3_3770 protein | cgd3_3770 | 80.877 | 176.05 | 31.3 | 21 | 33 | | 21 |
| Q5CY21 | Cryptopsoridial mucin, large thr stretch, signal peptide sequence | cgd7_4020 | 202.6 | 173.26 | 18.9 | 30 | 61 | | 30 |
| Q7YYX6 | Uncharacterized protein | 1MB.834 | 142.26 | 171.95 | 24.6 | 30 | 39 | | 30 |
| A3FQR9 | Latent transforming growth factor beta binding protein like (3F865) (Fragment) | cgd3_190 | 67.718 | 171.65 | 27.1 | 15 | 45 | | 15 |
| Q5CYV1 | Phosphoglycerate kinase (Fragment) | cgd7_910 | 43.313 | 164.63 | 54.7 | 22 | 34 | | 22 |
| Q5CVN3 | Zincin/aminopeptidase N like metalloprotease | cgd8_3430 | 105.91 | 163.13 | 23.2 | 22 | 29 | | 22 |
| P26183 | Actin | --- | 42.174 | 162.82 | 50.8 | 15 | 50 | | 15 |
| Q23716 | Elongation factor 2 | --- | 92.76 | 161.14 | 27.3 | 19 | 34 | | 19 |
| Q5CXY4 | Elongation factor EF1-gamma (Glutathione S-transferase family) | cgd7_4450 | 43.088 | 159.4 | 37.7 | 14 | 19 | | 14 |
| Q9TW48 | Oocyst wall protein (Fragment) | --- | 12.323 | 155.72 | 59.3 | 5 | 57 | | 1 |
| F0X475 | Cgd4_1940 protein | cgd4_1940 | 16.818 | 150.75 | 78.7 | 12 | 49 | | 12 |
| A3FQI2 | MORN repeat protein | cgd5_1640 | 34.154 | 145.54 | 57.3 | 13 | 22 | | 13 |
| Q5UVY5 | Thioredoxin peroxidase-like protein | --- | 21.772 | 143.82 | 56.1 | 14 | 26 | | 14 |
| Q5CSY2 | Peptidyl-prolyl cis-trans isomerase | cgd1_870 | 22.871 | 143.47 | 68.6 | 11 | 26 | | 11 |
| Q5CRF0 | 40S ribosomal protein S7 (Fragment) | cgd5_3040 | 23.05 | 139.64 | 57.8 | 14 | 19 | | 14 |
| Q5CQV2 | 40S ribosomal protein S3a | cgd4_3160 | 29.788 | 128.7 | 58 | 16 | 26 | | 16 |
| Q5CUC0 | Amine oxidase | cgd3_3430 | 199.34 | 127.13 | 16.8 | 26 | 31 | | 26 |
| Q6S5J0 | Oocyst wall protein 4 | COWP4 | 100 | 125.36 | 15.4 | 11 | 24 | | 11 |
| Q86PQ7 | TSP1 domain-containing protein TSP3 | --- | 55.752 | 124.46 | 25.8 | 9 | 13 | | 9 |
| Q5CUK8 | Ribosomal protein S18A, rps18ap, HhH domain | cgd3_2440 | 17.542 | 123.61 | 43.8 | 8 | 11 | | 8 |
| Q7YY92 | Putative arabinogalactan protein, possible | 1MB.373 | 111.47 | 121.2 | 21.5 | 21 | 29 | | 21 |
| F0X617 | Cgd5_1580 protein | cgd5_1580 | 43.849 | 117.67 | 40.5 | 17 | 30 | | 17 |
| Q5CSE9 | Fructose-bisphosphate aldolase (Fragment) | cgd1_3020 | 39.968 | 117.01 | 53.7 | 18 | 25 | | 18 |
| Q5CQ70 | Calmodulin-like protein | cgd5_3920 | 25.059 | 116.13 | 27.7 | 5 | 5 | | 5 |
| F0X3K6 | Cgd5_1960 protein | cgd5_1960 | 48.45 | 114.72 | 37 | 16 | 26 | | 16 |
| Q5CV61 | Signal peptide containing protein | cgd8_5300 | 90.315 | 114.02 | 23.2 | 13 | 17 | | 13 |
| Q9XYS7 | Cgd8_400 protein | cgd8_400 | 21.401 | 109.98 | 52.6 | 12 | 37 | | 12 |
| Q5CYZ8 | Uncharacterized protein | cgd7_400 | 38.579 | 108.01 | 44.4 | 13 | 34 | | 13 |
| Q5CTG6 | Uncharacterized protein | cgd2_3090 | 56.971 | 103.83 | 29.1 | 10 | 12 | | 10 |
| F0X6C0 | Cgd7_2280 protein | cgd7_2280 | 14.667 | 103.82 | 36.7 | 5 | 13 | | 5 |
| Q5CSZ9 | Predicted secreted protein, signal peptide | cgd1_660 | 37.102 | 103.7 | 14.5 | 5 | 13 | | 5 |
| Q5CS98 | Myosin regulatory light chain | cgd1_3570 | 30.146 | 103.13 | 33.3 | 8 | 12 | | 8 |
| Q5CS78 | Domain KOG1015, transcription regulator XNP/ATRX, DEAD-box superfamily, signal peptide | cgd1_3780 | 92.017 | 99.729 | 29.6 | 21 | 24 | | 21 |
| Q5CY39 | Unconventional myosin | cgd7_3790 | 91.479 | 97.632 | 30.1 | 25 | 35 | | 25 |
| Q94437 | Cgd4_3620 protein | p23 | 11.232 | 97.485 | 67.6 | 5 | 10 | | 5 |
| Q5CYJ3 | 60S ribosomal proteins L8/L2 | cgd7_2110 | 28.152 | 96.168 | 49 | 10 | 21 | | 10 |
| F0X4H0 | 40S ribosomal protein S6 | cgd2_4260 | 25.567 | 94.068 | 35.7 | 8 | 11 | | 8 |
| Q5CY94 | Uncharacterized protein | cgd7_3210 | 35.31 | 93.813 | 24.2 | 5 | 6 | | 5 |
| Q7YY89 | Uncharacterized protein | 1MB.378 | 72.484 | 89.958 | 21.2 | 13 | 16 | | 13 |
| Q5CUW3 | Ribosomal protein L14 (Fragment) | cgd3_1250 | 14.322 | 89.465 | 54.4 | 8 | 12 | | 8 |
| Q5CTQ4 | Uncharacterized protein | cgd2_2100 | 19.898 | 87.941 | 7.3 | 1 | 1 | | 1 |
| F0X4M3 | 60S ribosomal protein L13 | cgd2_2990 | 23.762 | 86.146 | 37.7 | 9 | 15 | | 9 |
| Q5CPV5 | 60S ribosomal protein L23A (Fragment) | cgd8_650 | 17.549 | 86.12 | 39.7 | 6 | 8 | | 6 |
| A0A2H4U2H8 | COWP protein (Fragment) | COWP | 15.263 | 86.116 | 45.1 | 4 | 36 | | 1 |
| Q5CYM0 | Secreted UDP-N-acetylglucosamine pyrophosphorylase family protein, signal peptide | cgd7_1830 | 72.915 | 83.743 | 22.5 | 14 | 17 | | 14 |
| Q5CSS6 | Predicted secreted protein, signal peptide | cgd1_1510 | 31.194 | 83.556 | 27.4 | 7 | 10 | | 7 |
| Q5CYA6 | Uncharacterized protein | cgd7_3070 | 29.706 | 82.063 | 37.1 | 8 | 10 | | 8 |
| Q5CSM7 | Pyruvate kinase (Fragment) | cgd1_2040 | 57.172 | 79.345 | 29.3 | 13 | 23 | | 13 |
| O76468 | Proteasome subunit alpha type | cgd2_2050 | 29.346 | 78.745 | 20.5 | 5 | 5 | | 5 |
| Q8MPB3 | Tubulin beta chain | 1MB.716 | 49.839 | 78.172 | 24.1 | 9 | 10 | | 9 |
| Q5CRE1 | Histone H2B (Fragment) | cgd5_3170 | 16.812 | 77.661 | 42.1 | 7 | 9 | | 6 |
| Q5CRB7 | Nascent polypeptide associated complex alpha chain with an NAC domain | cgd5_3450 | 21.096 | 76.893 | 50 | 6 | 8 | | 6 |
| Q5CTE6 | APG-1 like HSP70 domain containing protein, signal peptide plus likely ER retention motif | cgd2_3330 | 103.32 | 76.223 | 15.5 | 13 | 17 | | 13 |
| Q5CX06 | Uncharacterized protein | cgd6_3020 | 48.923 | 76.15 | 22.7 | 7 | 10 | | 7 |
| Q5CYL4 | KH domain protein | cgd7_1890 | 91.251 | 75.36 | 22.6 | 18 | 19 | | 18 |
| Q7YZ49 | 40S ribosomal protein S8 | 1MB.687 | 23.497 | 74.76 | 46.2 | 8 | 15 | | 8 |
| Q5CVF1 | Uncharacterized protein | cgd8_4290 | 26.702 | 74.117 | 42 | 10 | 18 | | 10 |
| Q5CU79 | 60S ribosomal protein L30, pelota RNA binding domain containing protein (Fragment) | cgd3_3890 | 12.178 | 74.116 | 53.2 | 5 | 20 | | 5 |
| Q5CPN9 | 60S acidic ribosomal protein LP2 | cgd2_130 | 11.511 | 73.944 | 47.3 | 4 | 8 | | 4 |
| F0X3G6 | Cgd2_1870 protein | cgd2_1870 | 34.082 | 72.423 | 45.4 | 10 | 13 | | 10 |
| Q7YYH5 | Cgd6_2170 protein | cgd6_2170 | 19.635 | 72.12 | 30.2 | 6 | 11 | | 6 |
| F0X3J6 | Cgd6_3190 protein | cgd6_3190 | 43.065 | 70.312 | 40.3 | 19 | 30 | | 19 |
| Q5CU33 | Uncharacterized protein | cgd2_490 | 43.433 | 69.652 | 22.5 | 10 | 15 | | 10 |
| Q9NJV7 | Proteasome subunit beta | PB | 22.962 | 69.31 | 33.3 | 8 | 8 | | 8 |
| F0X3S0 | Cgd8_440 protein | cgd8_440 | 35.376 | 68.906 | 22.8 | 6 | 11 | | 6 |
| Q5UVY7 | Superoxide dismutase | --- | 25.471 | 68.249 | 18.6 | 3 | 6 | | 3 |
| Q7YYY4 | Putative ABC transporter protein, possible | 1MB.816 | 86.397 | 68.059 | 10.2 | 8 | 9 | | 8 |
| Q5CQU4 | Articulin family protein, adjacent paralogous protein | cgd4_3290 | 58.255 | 67.935 | 13 | 6 | 9 | | 6 |
| Q5CVE2 | Uncharacterized protein | cgd8_4390 | 19.288 | 67.677 | 27.1 | 5 | 10 | | 5 |
| Q7YYG0 | Uncharacterized protein | 1MB.250 | 31.842 | 65.464 | 28.5 | 6 | 6 | | 6 |
| Q5CQG2 | Putative 60S ribosomal protein L24 | cgd4_840 | 15.279 | 61.79 | 27.8 | 4 | 9 | | 4 |
| Q5CQN4 | APG7-like ubiquitin activating enzyme E1 | cgd4_40 | 64.97 | 58.931 | 18.5 | 9 | 11 | | 9 |
| Q7YY51 | Similar to glycogen debranching enzyme, possible | 1MB.26 | 217.68 | 58.837 | 9.9 | 14 | 16 | | 14 |
| Q5CVY2 | Cold shock RNA binding domain of the OB fold (Fragment) | cgd8_2340 | 15.054 | 58.786 | 37.8 | 5 | 10 | | 5 |
| Q7YYT9 | Uncharacterized protein | 1MB.466 | 22.569 | 58.228 | 27.1 | 3 | 3 | | 3 |
| Q5CYH4 | SAR1-like small GTpase (Fragment) | cgd7_2330 | 24.135 | 58.019 | 28 | 5 | 7 | | 5 |
| Q5CQU3 | Articulin family protein, adjacent paralogous protein | cgd4_3300 | 30.076 | 57.877 | 18.6 | 5 | 7 | | 5 |
| Q5CUT6 | Large protein with signal peptide. cysteine-rich, threonine-rich, possible mucin | cgd3_1540 | 184.65 | 57.537 | 12.9 | 18 | 18 | | 18 |
| Q7YYE9 | Ribosomal protein L21, probable | 1MB.268 | 17.882 | 56.651 | 35.2 | 6 | 13 | | 6 |
| A3FQ01 | Uncharacterized protein | cgd8_4310 | 22.693 | 56.145 | 9.8 | 2 | 8 | | 2 |
| Q5CYU7 | Putative Sec61 signal peptide plus 9 transmembrane domain-containing protein | cgd7_950 | 52.376 | 54.891 | 12.7 | 7 | 10 | | 7 |
| Q5CWL3 | 60S ribosomal protein L26 (Fragment) | cgd6_4620 | 15.387 | 54.712 | 51.1 | 10 | 18 | | 10 |
| F0X4E3 | Cgd7_130 protein | cgd7_130 | 16.101 | 54.671 | 50 | 9 | 17 | | 9 |
| Q5CV30 | Putative fucose translocator with 8 transmembrane domains, within locus of 3 paralogous genes | cgd3_490 | 46.79 | 54.64 | 11.8 | 5 | 9 | | 5 |
| Q5CXN5 | Serine/threonine rich low complexity protein | cgd7_5520 | 102.12 | 54.54 | 12 | 9 | 10 | | 9 |
| F0X3W7 | 60S acidic ribosomal protein P0 | cgd4_2260 | 33.453 | 54.162 | 23.2 | 8 | 10 | | 8 |
| Q7YY44 | CRAL/TRIO cell signalling protein, possible | 1MB.41 | 49.76 | 53.884 | 26.1 | 10 | 11 | | 10 |
| Q5CTJ8 | Brf1p like coiled coil protein | cgd2_2700 | 57.188 | 52.088 | 31.3 | 15 | 22 | | 15 |
| Q5CUS3 | Uncharacterized protein | cgd3_1690 | 37.104 | 51.783 | 10 | 3 | 5 | | 3 |
| Q5CT88 | Protein with signal peptide, ER retention motif and 4x EF hands | cgd2_3990 | 51.328 | 51.748 | 17.4 | 6 | 6 | | 6 |
| Q5CTF8 | Glucose-6-phosphate isomerase | cgd2_3200 | 63.211 | 51.097 | 15.3 | 8 | 10 | | 8 |
| Q5CUU9 | Pyrophosphate--fructose 6-phosphate 1-phosphotransferase | cgd3_1400 | 148.08 | 51.078 | 10.8 | 13 | 13 | | 13 |
| Q5CXZ9 | Phosphoglycerate mutase | cgd7_4270 | 28.267 | 50.885 | 36.1 | 10 | 13 | | 10 |
| Q5CSF1 | 60S ribosomal protein L27 (Fragment) | cgd1_3000 | 18.549 | 50.83 | 24.7 | 5 | 12 | | 5 |
| Q5CY06 | Cation-transporting P-type ATpase with 11 or more transmembrane domains | cgd7_4190 | 123.97 | 50.814 | 8.4 | 9 | 10 | | 9 |
| A3FQ42 | Uncharacterized protein | cgd3_3200 | 43.589 | 50.603 | 30.7 | 10 | 11 | | 10 |
| Q5CQ89 | 40S ribosomal protein S17 | cgd5_3720 | 14.975 | 50.362 | 16.8 | 6 | 8 | | 6 |
| A3FQB9 | Dynamin-like protein | cgd1_580 | 84.49 | 50.046 | 6.6 | 3 | 3 | | 3 |
| Q5CQS1 | Secreted protein with signal peptide and 12 KAZAL repeats and a mucin-like stretch of threonines | cgd4_3550 | 136.7 | 49.98 | 11.2 | 10 | 10 | | 10 |
| Q7YXY2 | p23 co-chaperone, probable | 1MB.139 | 21.195 | 49.691 | 19.2 | 3 | 3 | | 3 |
| Q5CWE0 | Uncharacterized protein | cgd8_690 | 76.733 | 49.476 | 1.4 | 1 | 1 | | 1 |
| Q5CPY2 | Asparaginyl-tRNA synthetase (NOB+tRNA synthase) | cgd8_350 | 56.203 | 48.978 | 23.2 | 14 | 14 | | 14 |
| Q5CQ67 | Ran-binding protein | cgd5_3950 | 25.47 | 48.367 | 28 | 6 | 7 | | 6 |
| Q5CUB2 | Uncharacterized protein | cgd3_3510 | 13.817 | 48.352 | 42.1 | 6 | 8 | | 6 |
| Q5CYL3 | Mitochondrial NADH dehydrogenase | cgd7_1900 | 64.18 | 48.232 | 18.7 | 11 | 12 | | 11 |
| Q5CYF4 | 60S ribosomal protein L35A | cgd7_2540 | 13.556 | 48.186 | 46.7 | 7 | 17 | | 7 |
| Q5CUS1 | Uncharacterized protein | cgd3_1710 | 34.132 | 47.34 | 16 | 5 | 5 | | 5 |
| A3FQM0 | Heat shock 105kD heat shock 105kD alpha heat shock 105kD beta heat shock 105kDa protein 1 | cgd4_3270 | 90.709 | 46.771 | 17.4 | 12 | 16 | | 12 |
| Q5CVE6 | 60S ribosomal protein L7 | cgd8_4350 | 29.062 | 46.428 | 38.2 | 9 | 21 | | 9 |
| Q5CTQ3 | Uncharacterized protein | cgd2_2110 | 20.291 | 45.749 | 29.8 | 4 | 8 | | 4 |
| F0X3T7 | Cgd3_3040 protein | cgd3_3040 | 45.452 | 45.548 | 11 | 4 | 4 | | 4 |
| F0X4G1 | Cgd5_970 protein | cgd5_970 | 22.728 | 45.439 | 36.1 | 8 | 10 | | 8 |
| Q5CYI1 | 40S ribosomal protein S3, KH domain | cgd7_2250 | 24.851 | 45.399 | 43 | 10 | 16 | | 10 |
| F0X3L6 | Cgd3_330 protein | cgd3_330 | 30.663 | 45.335 | 30.3 | 7 | 8 | | 7 |
| Q5CRS5 | Uncharacterized protein | cgd5_1670 | 16.8 | 45.119 | 21.7 | 3 | 5 | | 3 |
| Q5CV68 | Histone H4 | cgd8_5230 | 11.31 | 44.967 | 28.2 | 3 | 5 | | 3 |
| Q5CQ52 | Possible EF hand protein | cgd5_4110 | 20.815 | 44.821 | 40.1 | 7 | 8 | | 7 |
| Q5CTH5 | 40S ribosomal protein S16 | cgd2_3000 | 16.096 | 44.7 | 40.3 | 7 | 13 | | 7 |
| Q5CQ27 | Uncharacterized protein | cgd5_4370 | 90.173 | 44.383 | 15.1 | 10 | 11 | | 10 |
| Q7YYP7 | Hypothetical garp protein, possible | 1MB.535 | 91.027 | 44.061 | 10.9 | 8 | 8 | | 8 |
| Q5CTQ2 | High mobility group small protein (Fragment) | cgd2_2120 | 11.138 | 43.884 | 31.6 | 4 | 5 | | 4 |
| Q7YY96 | Polyadenylate-binding protein | 1MB.364 | 81.073 | 42.871 | 13.5 | 8 | 9 | | 8 |
| Q5CV84 | Articulin family protein, Pfs77 protein-related | cgd8_5030 | 36.76 | 42.261 | 35.8 | 12 | 17 | | 12 |
| Q5CVF0 | Uncharacterized protein | cgd8_4300 | 19.66 | 41.969 | 37.9 | 5 | 10 | | 5 |
| A3FQM8 | Uncharacterized protein (Fragment) | cgd4_32 | 55.911 | 41.916 | 16.9 | 8 | 8 | | 8 |
| Q5CQW8 | Dbp1p, eIF4a-1 family RNA SFII helicase (DEXDC+HELICc) | cgd4_3000 | 78.713 | 41.704 | 6.3 | 4 | 5 | | 4 |
| F0X3H7 | Cgd5_2800 protein | cgd5_2800 | 15.543 | 41.031 | 37.3 | 5 | 11 | | 5 |
| Q5CRR4 | MJ1625/yease Yp1009cp-like HhH domain (Fragment) | cgd5_1780 | 157.91 | 41.009 | 4.4 | 5 | 5 | | 5 |
| Q5CTR8 | Putative phosphatidylinositol-4-phosphate 5-kinase, MORN beta hairpin repeats glycine-rich protein | cgd2_1910 | 60.445 | 40.918 | 15.9 | 8 | 9 | | 8 |
| Q9GT92 | Lactate dehydrogenase | LDH1 | 33.905 | 40.831 | 35.5 | 10 | 13 | | 10 |
| F0X611 | Cgd7_1680 protein | cgd7_1680 | 24.145 | 40.574 | 26.5 | 5 | 6 | | 5 |
| Q5CPW2 | Proteasome regulatory subunit S2 (RPN1) (Fragment) | cgd8_570 | 114.62 | 40.484 | 8.3 | 7 | 9 | | 7 |
| F0X4J6 | Cgd7_5000 protein | cgd7_5000 | 67.596 | 40.427 | 24.2 | 14 | 14 | | 14 |
| Q5CVB3 | Seryl-tRNA synthetase, cytoplasmic | cgd8_4720 | 52.063 | 40.286 | 13.7 | 5 | 6 | | 5 |
| Q5CUA4 | Possible aspartyl aminopeptidase (Fragment) | cgd3_3610 | 52.432 | 40.28 | 31.2 | 12 | 15 | | 12 |
| Q5CRD6 | Proteasome subunit beta7 NTN hydrolase fold | cgd5_3220 | 35.359 | 39.893 | 27.1 | 8 | 8 | | 8 |
| Q5CQP2 | T-complex protein 1 subunit gamma (Fragment) | cgd4_3850 | 61.122 | 39.892 | 10.7 | 6 | 6 | | 6 |
| F0X4K7 | Proteasome subunit alpha type | cgd4_250 | 29.098 | 39.88 | 25.7 | 5 | 5 | | 5 |
| Q5CQ08 | Uncharacterized protein | cgd8_70 | 48.468 | 39.686 | 17.5 | 6 | 16 | | 6 |
| Q7YZ16 | Cgd6_4950 protein | cgd6_4950 | 30.382 | 39.636 | 27.1 | 6 | 7 | | 6 |
| Q5CYW2 | Uncharacterized protein (Fragment) | cgd7_790 | 13.808 | 39.56 | 16 | 1 | 1 | | 1 |
| Q5CY16 | Protein disulfide isomerase, signal peptide, ER retention motif (Fragment) | cgd7_4080 | 50.515 | 39.309 | 21.7 | 10 | 14 | | 10 |
| Q5CT04 | Uncharacterized protein | cgd1_590 | 281.05 | 39.286 | 4 | 10 | 10 | | 10 |
| A3FPR5 | Ribosomal protein S4, putative (Fragment) | cgd7_4760 | 28.987 | 38.855 | 45.8 | 13 | 24 | | 13 |
| Q5CXM9 | Uncharacterized protein | cgd6_420 | 43.462 | 38.77 | 9 | 3 | 4 | | 3 |
| Q5CXL5 | 60S ribosomal protein L23 (Fragment) | cgd6_570 | 15.564 | 38.307 | 39.7 | 6 | 14 | | 6 |
| Q5CYT5 | Uncharacterized protein | cgd7_1090 | 43.992 | 38.162 | 8.8 | 3 | 3 | | 3 |
| A3FQI4 | Proteasome subunit alpha type (Fragment) | cgd5_1820 | 28.102 | 37.99 | 28.6 | 8 | 8 | | 8 |
| A4L7M9 | 15 kDa sporozoite surface antigen (Fragment) | CP15 | 13.259 | 37.988 | 37.8 | 4 | 8 | | 4 |
| Q5CR33 | Ubiquitin-activating enzyme E1 (UBA) (Fragment) | cgd4_2300 | 120.52 | 37.925 | 8.2 | 9 | 10 | | 9 |
| Q7YYK8 | Cgd6_4320 protein | cgd6_4320 | 21.824 | 37.87 | 52 | 13 | 17 | | 13 |
| Q27549 | Acetyl-coenzyme A synthetase | ACS | 77.97 | 37.849 | 13.7 | 8 | 8 | | 8 |
| Q5CU08 | Uncharacterized protein | cgd2_770 | 50.453 | 37.81 | 10.2 | 4 | 6 | | 4 |
| Q5CYH9 | Hypothetical low complexity protein | cgd7_2270 | 40.646 | 37.322 | 10.2 | 3 | 3 | | 3 |
| Q5CVT9 | Extracellular protein with a signal peptide, a mucin-like stretch of threonine residues and 6 notch domains possible mucin | cgd8_2800 | 65.315 | 37.214 | 9.7 | 5 | 6 | | 5 |
| Q5CU23 | T-complex protein 1, alpha subunit | cgd2_600 | 61.546 | 37.133 | 21 | 11 | 11 | | 11 |
| Q5CUZ6 | ARF1/2 like small GTpase (Fragment) | cgd3_860 | 21.456 | 37.074 | 39.7 | 7 | 10 | | 4 |
| F0X506 | Cgd8_1670 protein | cgd8_1670 | 55.175 | 37.064 | 17.8 | 7 | 7 | | 7 |
| Q5CYP8 | Tryptophanyl-tRNA synthetase | cgd7_1490 | 67.885 | 36.887 | 8.9 | 6 | 6 | | 6 |
| Q7YY41 | Uncharacterized protein | 1MB.45 | 55.582 | 36.74 | 3 | 1 | 1 | | 1 |
| Q7YY57 | Uncharacterized protein | 1MB.11 | 34.122 | 36.732 | 17.8 | 4 | 9 | | 4 |
| Q5CUS6 | eIF2G GTpase. eukaryotic translation initiation factor 2 gamma subunit (ZnR+GTpase) (Fragment) | cgd3_1650 | 52.148 | 36.725 | 21.3 | 9 | 10 | | 9 |
| Q5CY51 | Proteasome subunit alpha type (Fragment) | cgd7_3660 | 31.716 | 36.677 | 11.3 | 3 | 4 | | 3 |
| Q5CXW4 | Hypothetical secreted protein | cgd7_4680 | 44.829 | 36.457 | 15.5 | 4 | 5 | | 4 |
| F0X4X8 | Cgd8_3290 protein | cgd8_3290 | 38.257 | 35.877 | 15.7 | 6 | 8 | | 6 |
| Q5CSM0 | Glutaminyl-tRNA synthetase, of predicted bacterial origin | cgd1_2130 | 74.957 | 35.457 | 14.4 | 9 | 12 | | 9 |
| Q5CUM9 | Proteasome endopeptidase complex | cgd3_2170 | 28.043 | 35.256 | 34.1 | 8 | 8 | | 8 |
| A3FQ78 | RAB6 protein, putative | cgd2_1940 | 25.185 | 34.838 | 13.7 | 3 | 3 | | 3 |
| Q5CQZ5 | P-type ATpase involved in cation transport | cgd4_2720 | 168.83 | 34.652 | 5.3 | 7 | 7 | | 7 |
| Q5CS32 | Protein with 2 CAP (CARP) domains, possible adenyl cyclase-associated protein (Fragment) | cgd5_440 | 20.428 | 34.627 | 51.9 | 9 | 12 | | 9 |
| Q5CTH0 | Uncharacterized protein | cgd2_3050 | 15.745 | 34.523 | 24.6 | 3 | 3 | | 3 |
| Q5CTI4 | 40S ribosomal protein S28, no good start Met (Fragment) | cgd2_2870 | 7.4996 | 34.477 | 47.1 | 3 | 6 | | 3 |
| Q5CTX4 | Steroid reductase like intregral membrane protein with 4x transmembrane domains and an ubiquitin domain at its N-terminus | cgd2_1200 | 36.346 | 34.416 | 8.7 | 3 | 4 | | 3 |
| Q5CT92 | Putative translation elongation factor 1 beta 1 | cgd2_3950 | 27.618 | 34.34 | 25.9 | 7 | 14 | | 7 |
| Q86PS0 | Cgd1_3530 protein | GBP | 22.985 | 34.326 | 26.3 | 4 | 5 | | 4 |
| Q5CT24 | CDC48 like AAA ATPase ortholog (Fragment) | cgd1_330 | 90.56 | 34.257 | 13.8 | 11 | 11 | | 11 |
| Q7YZ69 | Aminoacyl-tRNA synthetase, probable | 1MB.635 | 79.168 | 34.15 | 14.8 | 9 | 10 | | 9 |
| F0X3P1 | Profilin | cgd3_1570 | 17.606 | 34.085 | 11.7 | 2 | 5 | | 2 |
| Q5CTL2 | Uncharacterized protein | cgd2_2550 | 216.71 | 33.757 | 4.6 | 9 | 10 | | 9 |
| Q5CRM9 | Nuc1p like endonuclease G | cgd5_2190 | 53.875 | 33.442 | 7.2 | 3 | 3 | | 3 |
| Q7YZ06 | Dolichyl-diphosphooligosaccharide--protein glycosyltransferase subunit 1 | 1MB.766 | 55.895 | 33.329 | 12.6 | 5 | 7 | | 5 |
| Q6XLN2 | Putative fatty acid long chain acyl-CoA ligase | ACL1 | 78.181 | 32.903 | 13.3 | 8 | 8 | | 8 |
| A3FQL3 | UDP-glucose 4-epimerase | cgd4_2600 | 38.571 | 32.623 | 27.5 | 8 | 11 | | 8 |
| Q5CTS2 | PDI like thioredoxin domain containing protein | cgd2_1780 | 49.734 | 32.599 | 11.6 | 5 | 5 | | 5 |
| Q5CR81 | Uncharacterized protein | cgd4_1770 | 27.424 | 32.437 | 23.6 | 5 | 9 | | 5 |
| Q5CQA5 | V-type proton ATPase subunit a (Fragment) | cgd4_1470 | 104.81 | 32.411 | 4.6 | 4 | 4 | | 4 |
| Q5CTG4 | Uncharacterized protein | cgd2_3110 | 115.03 | 32.219 | 9.2 | 9 | 9 | | 9 |
| Q5CTF6 | Sc6p-like, SM domain | cgd2_3220 | 40.081 | 32.161 | 16.4 | 6 | 6 | | 6 |
| Q5CR10 | 26S proteasome regulatory subunit 26b like AAA ATpase | cgd4_2540 | 45.484 | 32.145 | 12.2 | 4 | 4 | | 4 |
| Q5CTY4 | 40S ribosomal protein S25 | cgd2_1070 | 11.596 | 31.944 | 52.8 | 5 | 8 | | 5 |
| Q5CUP3 | PP2C like protein phosphatase (Fragment) | cgd3_2020 | 71.132 | 31.825 | 12.7 | 6 | 6 | | 6 |
| F0X429 | Cgd8_2870 protein | cgd8_2870 | 24.428 | 31.824 | 22 | 7 | 9 | | 7 |
| Q7YYC7 | Peptidylprolyl isomerase | 1MB.312 | 36.814 | 31.751 | 21.5 | 6 | 7 | | 6 |
| Q5CXZ5 | Extracellular protein with a signal peptide and 4 SCP domains | cgd7_4310 | 184.95 | 31.695 | 2 | 3 | 4 | | 3 |
| F0X3S2 | Cgd7_5060 protein | cgd7_5060 | 14.284 | 31.458 | 30.7 | 4 | 6 | | 4 |
| Q5CVX7 | Sey1p like P-loop GTpase of the RHD3 subfamily with a transmembrane domain near C-terminus (Fragment) | cgd8_2410 | 97.158 | 31.366 | 5 | 4 | 5 | | 4 |
| F0X4Z1 | Cgd7_4300 protein | cgd7_4300 | 39.122 | 31.33 | 18.6 | 5 | 6 | | 5 |
| Q7YZ54 | Cgd6_4570 protein | cgd6_4570 | 54.314 | 31.201 | 20.8 | 10 | 10 | | 10 |
| W0C8U4 | Oocyst wall protein (Fragment) | COWP | 21.171 | 31.161 | 72.6 | 9 | 91 | | 1 |
| Q5CU88 | 60S ribosomal protein L19 (Fragment) | cgd3_3790 | 20.563 | 30.81 | 20.6 | 3 | 7 | | 3 |
| Q5CPE8 | Uncharacterized protein | cgd6_5500 | 34.168 | 30.705 | 24.1 | 7 | 8 | | 7 |
| Q5CTP3 | Proteasome regulatory subunit Rpn7/26S proteasome subunit 6, PINT domain containing protein | cgd2_2210 | 47.438 | 30.694 | 9.4 | 4 | 4 | | 4 |
| Q5CV48 | Rasputin. nuclear transport factor 2 (NTF2) domain plus RRM domain | cgd3_300 | 53.667 | 30.659 | 15.2 | 6 | 7 | | 6 |
| Q5CSE7 | Triosephosphate isomerase | cgd1_3040 | 27.384 | 30.503 | 33.2 | 6 | 9 | | 6 |
| Q5CQJ4 | 60S ribosomal protein L18 (Fragment) | cgd4_470 | 21.441 | 30.366 | 28.4 | 6 | 10 | | 6 |
| F0X5R3 | Cgd4_4020 protein | cgd4_4020 | 28.983 | 29.971 | 37.8 | 11 | 19 | | 11 |
| A3FQP6 | Uncharacterized protein | cgd5_4400 | 70.955 | 29.885 | 9.1 | 4 | 4 | | 4 |
| Q5CQ95 | Uncharacterized protein | cgd5_3650 | 35.001 | 29.723 | 28.9 | 6 | 7 | | 6 |
| F0X4I0 | GTP-binding nuclear protein | cgd7_220 | 24.109 | 29.361 | 30.7 | 7 | 8 | | 7 |
| Q7YZ70 | Uncharacterized protein | 1MB.633 | 65.396 | 29.303 | 9.9 | 4 | 5 | | 4 |
| Q5CQ14 | Uncharacterized protein | cgd8_10 | 33.195 | 28.791 | 35.2 | 9 | 10 | | 9 |
| A3FPY5 | 40S ribosomal subunit protein S9, putative | cgd8_1840 | 12.494 | 28.542 | 31.2 | 3 | 6 | | 3 |
| Q5CYR4 | Extracellular protein with a signal peptide followed by a family 2 glycosyltransferase and ricin domains (Fragment) | cgd7_1310 | 63.208 | 28.479 | 13 | 8 | 9 | | 8 |
| Q6VPE9 | Malate dehydrogenase | MalDH1 | 33.628 | 28.199 | 15.5 | 4 | 4 | | 4 |
| Q5CTB1 | Uncharacterized protein | cgd2_3730 | 29.862 | 27.946 | 22.8 | 5 | 5 | | 5 |
| A3FQI3 | Uncharacterized protein (Fragment) | cgd5_1650 | 19.645 | 27.942 | 22.2 | 4 | 9 | | 4 |
| Q5CUR5 | Uncharacterized protein | cgd3_1770 | 59.902 | 27.91 | 13.9 | 5 | 5 | | 5 |
| Q5CS40 | Signal peptide containing protein with threonine stretches,possible mucin | cgd5_340 | 35.863 | 27.822 | 8.1 | 2 | 4 | | 2 |
| Q5CQH4 | Conserved protein of possible plant or bacterial origin (Fragment) | cgd4_700 | 47.153 | 27.178 | 13.1 | 5 | 10 | | 5 |
| F0X4T8 | Cgd5_2600 protein | cgd5_2600 | 58.002 | 27.083 | 14.7 | 8 | 12 | | 8 |
| F0X5E9 | Cgd3_2110 protein | cgd3_2110 | 51.812 | 26.766 | 11.8 | 4 | 4 | | 4 |
| Q5CRM7 | 40S ribosomal protein S13 (Fragment) | cgd5_2210 | 17.507 | 26.762 | 29.2 | 4 | 5 | | 4 |
| F0X3Y9 | Cgd3_1300 protein | cgd3_1300 | 17.842 | 26.63 | 39.5 | 5 | 9 | | 5 |
| Q5CUW0 | 14-3-3 domain containing protein (Fragment) | cgd3_1290 | 30.899 | 26.609 | 27.5 | 8 | 14 | | 8 |
| F0X4A3 | Cgd5_750 protein | cgd5_750 | 37.104 | 26.302 | 15.5 | 5 | 7 | | 5 |
| Q5CTY7 | Proteasome regulatory subunit Rpn9, PINT domain | cgd2_1020 | 49.683 | 26.244 | 10.2 | 4 | 4 | | 4 |
| F0X4R1 | Eukaryotic translation initiation factor 5A | cgd7_2300 | 17.263 | 26.234 | 16.2 | 2 | 3 | | 2 |
| F0X4H1 | Cgd5_820 protein | cgd5_820 | 58.801 | 26.104 | 6.6 | 3 | 3 | | 3 |
| Q5CRL6 | Actin-like | cgd5_2330 | 43.689 | 25.796 | 8.9 | 3 | 3 | | 3 |
| Q5CVM4 | Secreted protein with cysteine rich repeats and a mucin like threonine rich repeat, signal peptide | cgd8_3520 | 120.83 | 25.708 | 8.1 | 7 | 7 | | 7 |
| A3FQ97 | Heat shock protein DnaJ Pfj2, putative | cgd2_3230 | 67.32 | 25.094 | 3.3 | 2 | 2 | | 2 |
| Q5CPM4 | Ribosomal protein L15 | cgd2_280 | 24.18 | 25.002 | 32.7 | 7 | 11 | | 7 |
| Q5CT29 | Short chain dehydrogenase/reductase of the rossmann fold, signal peptide | cgd1_280 | 41.124 | 24.901 | 15.5 | 5 | 7 | | 5 |
| Q5CT75 | Articulin family protein | cgd2_4150 | 43.706 | 24.859 | 7.4 | 3 | 3 | | 3 |
| Q5CXK6 | Protein with signal peptide plus Thr stretch, possible mucin | cgd6_710 | 49.754 | 24.339 | 11.8 | 5 | 9 | | 5 |
| Q5CTL4 | Uncharacterized protein | cgd2_2530 | 38.968 | 24.24 | 15.2 | 5 | 5 | | 5 |
| Q5CXX9 | Sgnal peptide, large secreted protein | cgd7_4500 | 91.716 | 24.175 | 3.3 | 2 | 4 | | 2 |
| F0X473 | Cgd8_500 protein | cgd8_500 | 15.901 | 23.812 | 66 | 7 | 8 | | 7 |
| Q5CVH2 | 26S proteasomal subunit S3 PINT domain containing protein (Fragment) | cgd8_4060 | 63.488 | 23.799 | 5.6 | 3 | 4 | | 3 |
| Q5CQW0 | Ribosomal protein S27a, ubiquitin plus zincribbon, UB13p (Fragment) | cgd4_3080 | 17.867 | 23.474 | 21.4 | 3 | 4 | | 3 |
| Q7YYS5 | Uncharacterized protein | 1MB.491 | 74.955 | 23.3 | 6.9 | 3 | 3 | | 3 |
| K9MGK8 | Peptidyl-prolyl cis-trans isomerase | --- | 18.544 | 23.279 | 23.8 | 5 | 9 | | 5 |
| Q5CQY9 | DNAj domain protein having a signal peptide (Fragment) | cgd4_2780 | 40.786 | 23.191 | 13.6 | 3 | 3 | | 3 |
| Q7YYR4 | 1,4-alpha-glucan branching enzyme, possible | 1MB.513 | 74.559 | 23.001 | 3.1 | 2 | 2 | | 2 |
| Q5CXZ3 | Glycoprotein, putative | cgd7_4330 | 63.913 | 22.999 | 15.8 | 8 | 8 | | 8 |
| Q5CTL6 | Uncharacterized protein | cgd2_2510 | 85.172 | 22.91 | 6.3 | 4 | 4 | | 4 |
| Q5CUW6 | Uncharacterized protein | cgd3_1220 | 32.659 | 22.769 | 30.3 | 5 | 5 | | 5 |
| Q5CV33 | Glutathione peroxidase | cgd3_460 | 24.747 | 22.697 | 31.2 | 7 | 9 | | 7 |
| Q7YY77 | CAAX prenyl protease, possible | 56k.07 | 49.976 | 22.508 | 11.3 | 5 | 7 | | 5 |
| Q5CRU5 | Large protein with signal peptide | cgd5_1440 | 187.25 | 21.885 | 2.7 | 4 | 4 | | 4 |
| A2T125 | COWP protein (Fragment) | COWP | 25.193 | 21.878 | 43 | 8 | 96 | | 1 |
| Q7YYM4 | Myosin light chain, possible | 1MB.593 | 40.384 | 21.663 | 11.3 | 4 | 4 | | 4 |
| F0X4M5 | Nucleoside diphosphate kinase | cgd5_1470 | 24.924 | 21.525 | 11.7 | 3 | 7 | | 3 |
| A3FQ99 | ARF1/2 like small GTpase, putative | cgd2_3410 | 20.701 | 21.397 | 35.7 | 6 | 9 | | 3 |
| Q5CS35 | Putative eukaryotic translation initiation factor 3 | cgd5_390 | 120.43 | 21.047 | 3.8 | 4 | 4 | | 4 |
| O02494 | Eukaryotic initiation factor 4A | EIF4-A | 45.933 | 20.947 | 13.1 | 4 | 4 | | 4 |
| Q7YY36 | Dentin sialophosphoprotein, possible | 1MB.56 | 62.05 | 20.874 | 9.4 | 3 | 3 | | 3 |
| Q5CS75 | Uncharacterized protein | cgd1_3810 | 102.44 | 20.548 | 7.1 | 6 | 6 | | 6 |
| Q5CVN7 | Splicing factor SRP40 like 2x RRM domains (Fragment) | cgd8_3370 | 47.928 | 20.398 | 6.5 | 2 | 2 | | 2 |
| Q5CXU7 | Signal peptide plus GPI anchored membrane protein | cgd7_4880 | 36.8 | 20.264 | 14 | 3 | 7 | | 3 |
| F0X3U3 | Cgd2_4320 protein | cgd2_4320 | 54.727 | 20.263 | 8.3 | 3 | 6 | | 3 |
| R4JBX6 | C-type lectin-like domain containing protein | Clec | 86.382 | 20.193 | 3.6 | 3 | 3 | | 3 |
| Q5CU19 | Possible conserved acid phosphatase | cgd2_640 | 37.636 | 20.126 | 3.2 | 1 | 2 | | 1 |
| Q5CSK8 | 40S ribosomal protein S26 | cgd1_2270 | 12.191 | 20.064 | 29 | 3 | 6 | | 3 |
| Q7YYY9 | Uncharacterized protein | 1MB.803 | 123.7 | 19.86 | 10.7 | 9 | 9 | | 9 |
| Q5CXU2 | Gpantothenate kinase 4, rnaseH fold | cgd7_4940 | 49.866 | 19.824 | 13.1 | 5 | 5 | | 5 |
| Q5CYM2 | ALG1 like beta-1,4 mannosyltransferase with possible signal peptide | cgd7_1810 | 77.277 | 19.554 | 6.6 | 5 | 5 | | 5 |
| Q7YYJ9 | Cysteine protease inhibitor | 1MB.180 | 19.785 | 19.461 | 27 | 3 | 3 | | 3 |
| Q5CW33 | Uncharacterized protein | cgd8_1800 | 40.856 | 19.432 | 13.7 | 4 | 4 | | 4 |
| Q5CY61 | Uncharacterized protein | cgd7_3560 | 22.962 | 19.396 | 5.1 | 1 | 1 | | 1 |
| Q5CQL4 | WD repeat protein | cgd4_260 | 148.68 | 19.26 | 2.1 | 3 | 3 | | 3 |
| A3FQN1 | Rab11, putative | cgd4_320 | 24.746 | 19.058 | 19.5 | 4 | 4 | | 4 |
| Q5CQP1 | T-complex protein 1 subunit eta (Fragment) | cgd4_3860 | 62.032 | 18.885 | 5.1 | 3 | 3 | | 3 |
| Q5CYN3 | Nuclear pore protein RBP16/17 (RAN binding protein 16/17) (Fragment) | cgd7_1670 | 127.71 | 18.83 | 6.4 | 7 | 7 | | 7 |
| Q5CQV5 | Eukaryotic translation initiation factor 6 | EIF6 | 27.397 | 18.809 | 22.2 | 5 | 7 | | 5 |
| Q5CTV2 | Proteasome subunit alpha type (Fragment) | cgd2_1440 | 28.362 | 18.777 | 15.6 | 5 | 7 | | 5 |
| Q7YYF2 | Nascent polypeptide-associated complex subunit beta | 1MB.263 | 21.241 | 18.757 | 26.9 | 6 | 7 | | 6 |
| Q5CUM2 | 60S ribosomal protein L37A (Fragment) | cgd3_2250 | 9.5501 | 18.481 | 29.9 | 3 | 4 | | 3 |
| Q5CU34 | Uncharacterized protein | cgd2_480 | 52.385 | 18.442 | 9.1 | 4 | 4 | | 4 |
| Q5CY30 | DNAJ domain protein sec63 ortholog, 4 transmembrane domains | cgd7_3880 | 71.592 | 18.319 | 11.8 | 6 | 7 | | 6 |
| Q5CXN4 | Uncharacterized protein | cgd7_5530 | 11.409 | 18.258 | 8.9 | 1 | 1 | | 1 |
| Q5CVY3 | Pyridine nucleotide/ NAD(P) transhydrogenase alpha plus beta subunits, duplicated gene, possible signal peptide plus 12 transmembrane regions | cgd8_2330 | 123.78 | 18.145 | 9.3 | 8 | 8 | | 8 |
| Q5CTF2 | Phosphoglucomutase, tandemly duplicated protein | cgd2_3270 | 74.826 | 18.14 | 12.1 | 7 | 7 | | 5 |
| Q7YXZ9 | Galactosyltransferase, possible | 1MB.107 | 95.152 | 18.039 | 5.7 | 5 | 8 | | 5 |
| Q5CYQ6 | Signal peptide containing protein | cgd7_1400 | 79.115 | 17.974 | 7.1 | 5 | 5 | | 5 |
| Q5CW43 | NAD dependent dehydrogenase of possible bacterial origin (Fragment) | cgd8_1700 | 52.607 | 17.922 | 11 | 4 | 4 | | 4 |
| A3FPM4 | Putative patched family protein (Fragment) | cgd7_850 | 144.23 | 17.892 | 3.4 | 4 | 8 | | 4 |
| F0X3U0 | Proteasome subunit beta | cgd1_420 | 22.521 | 17.878 | 25.1 | 5 | 8 | | 5 |
| Q5CRN9 | SPAC694.02. SKI family SFII helicase | cgd5_2070 | 243.22 | 17.764 | 4.3 | 9 | 9 | | 9 |
| Q5CPD9 | Asparagine synthetase A (AsnA) like protein (Fragment) | cgd5_4540 | 40.447 | 17.629 | 13.4 | 4 | 5 | | 4 |
| Q5CWC6 | 26S proteasome regulatory subunit S10b like AAA+ ATpase (Fragment) | cgd8_840 | 45.543 | 17.556 | 13.8 | 6 | 9 | | 4 |
| Q5CPT7 | Rab GDP dissociation inhibitor (Fragment) | cgd4_4080 | 53.147 | 17.333 | 11 | 5 | 5 | | 5 |
| Q5CXQ4 | Thioredoxin/PDI, cyanobacterial type, signal peptide plus 4 transmembrane domains | cgd7_5330 | 75.574 | 17.246 | 4.7 | 2 | 2 | | 2 |
| Q06550 | Precursor of oocyst wall protein | --- | 175.67 | 16.988 | 60.1 | 78 | 727 | | 0 |
| Q5CR27 | Lysine--tRNA ligase | cgd4_2370 | 64.309 | 16.98 | 5.5 | 3 | 3 | | 3 |
| Q5CVP6 | DNAJ'DNAJ protein | cgd8_3270 | 69.328 | 16.639 | 5.6 | 3 | 3 | | 3 |
| Q5CYG5 | 60S ribosomal protein L22 | cgd7_2420 | 13.244 | 16.592 | 43.5 | 4 | 7 | | 4 |
| A3FPN1 | Aspartate--tRNA ligase | cgd7_1540 | 60.989 | 16.532 | 9.6 | 4 | 4 | | 4 |
| Q5CS64 | Phosphoenolpyruvate carboxylase | cgd5_70 | 130.83 | 16.52 | 5.4 | 6 | 8 | | 6 |
| Q5CQ43 | Proteasome subunit beta | cgd5_4210 | 32.522 | 16.516 | 15.9 | 5 | 5 | | 5 |
| Q5CTZ5 | Peptidase'insulinase-like peptidase | cgd2_930 | 116.23 | 16.374 | 3.4 | 3 | 3 | | 3 |
| Q7YYW6 | Small GTP binding protein rab1a, probable | 1MB.413 | 22.804 | 16.218 | 25.6 | 6 | 9 | | 6 |
| Q5CQS9 | Uncharacterized protein | cgd4_3470 | 49.617 | 16.172 | 8.4 | 4 | 4 | | 4 |
| Q5CVN1 | 60S ribosomal protein L17 | cgd8_3450 | 21.413 | 16.153 | 38 | 7 | 8 | | 7 |
| Q5CT41 | Uncharacterized protein | cgd1_140 | 110.97 | 16.022 | 2.1 | 2 | 2 | | 2 |
| A3FQC1 | Uncharacterized protein | cgd1_620 | 50.779 | 15.985 | 10.5 | 4 | 5 | | 4 |
| Q5CQC8 | 60S ribosomal protein L28 | cgd4_1230 | 14.415 | 15.982 | 23.6 | 3 | 8 | | 3 |
| Q7YYM1 | Cgd6_4190 protein | cgd6_4190 | 25.32 | 15.966 | 27.5 | 6 | 14 | | 6 |
| Q8I858 | Tubulin alpha chain (Fragment) | --- | 48.919 | 15.919 | 14.3 | 6 | 6 | | 6 |
| Q5CS69 | Signal peptide containing protein | cgd5_20 | 84.814 | 15.799 | 5.7 | 4 | 4 | | 4 |
| Q5CUM6 | Proteasome subunit alpha type | cgd3_2200 | 27.668 | 15.735 | 15.1 | 4 | 4 | | 4 |
| Q5CR75 | Dynein light chain tctex1 | cgd4_1830 | 16.561 | 15.695 | 10.6 | 1 | 1 | | 1 |
| Q7YYU3 | Cgd6_3470 protein | cgd6_3470 | 27.46 | 15.687 | 14 | 4 | 4 | | 4 |
| Q5CV74 | Uncharacterized apicomplexan-specific protein | cgd8_5160 | 18.892 | 15.632 | 7.6 | 1 | 1 | | 1 |
| Q7Z0D0 | S-adenosylmethionine synthase | SAMS | 44.841 | 15.192 | 9.9 | 4 | 4 | | 4 |
| A3FQ16 | Calmodulin-domain protein kinase 1, putative | cgd3_920 | 60.953 | 15.014 | 11.3 | 5 | 8 | | 5 |
| Q5CRS1 | CTP synthase | cgd5_1710 | 74.608 | 14.924 | 11.3 | 7 | 7 | | 7 |
| Q5CTF5 | ARF GAP-like zinc finger-containing protein (Fragment) | cgd2_3240 | 45.478 | 14.68 | 14.4 | 5 | 6 | | 5 |
| Q5CPU3 | Diamine acetyltransferase | SSAT | 17.837 | 14.578 | 18.4 | 3 | 3 | | 3 |
| Q5CV13 | Uncharacterized protein | cgd3_660 | 96.837 | 14.55 | 6.1 | 5 | 5 | | 5 |
| Q5CQ87 | 40S ribosomal protein S12 | cgd5_3740 | 15.835 | 14.503 | 45.5 | 5 | 8 | | 5 |
| Q5CUJ9 | Histone H3 | cgd3_2540 | 15.382 | 14.428 | 17.8 | 4 | 5 | | 4 |
| Q5CR28 | DSK2 like protein with a ubiquitin domain, 2 STI1 motifs and a UBA domain at its C-terminus (Fragment) | cgd4_2360 | 43.797 | 14.221 | 6.5 | 2 | 2 | | 2 |
| Q5CPU8 | RPN2/26s proteasome regulatory subunit | cgd4_3950 | 120.36 | 14.215 | 4.7 | 3 | 3 | | 3 |
| Q5CRV6 | Conserved protein with possible signal peptide and ER retention motif possible ER protein | cgd5_1310 | 31.213 | 14.194 | 14.8 | 4 | 4 | | 4 |
| Q5CW04 | Uncharacterized protein | cgd8_2110 | 25.412 | 14.122 | 22.2 | 4 | 4 | | 4 |
| A3FQ48 | Isoleucine-tRNA synthetase | cgd3_3840 | 128.76 | 13.92 | 5.6 | 6 | 6 | | 6 |
| Q5CRZ1 | Histone H2A | cgd5_940 | 15.682 | 13.88 | 26.3 | 4 | 6 | | 4 |
| Q5CYR8 | Uncharacterized protein | cgd7_1270 | 472.29 | 13.813 | 1.7 | 5 | 5 | | 5 |
| Q7YYZ4 | Conserved hypothetical transmembrane protein | 1MB.787 | 289.01 | 13.783 | 1.4 | 4 | 4 | | 4 |
| Q5CW94 | Coronin (Fragment) | cgd8_1180 | 93.246 | 13.674 | 6.1 | 5 | 5 | | 5 |
| Q5CZ18 | Uncharacterized protein | cgd7_180 | 16.863 | 13.607 | 34.9 | 5 | 11 | | 5 |
| Q5CXY3 | 60S ribosomal protein L6 | cgd7_4460 | 20.739 | 13.536 | 32.1 | 5 | 13 | | 5 |
| Q5CYE7 | ClpB ATpase (Bacterial), signal peptide | cgd7_2620 | 145.14 | 13.467 | 4.3 | 5 | 5 | | 5 |
| Q5CVE5 | 40S ribosomal protein S15A (Fragment) | cgd8_4360 | 15.642 | 13.462 | 30.7 | 5 | 8 | | 5 |
| Q5CUR6 | Uncharacterized protein | cgd3_1760 | 33.36 | 13.346 | 13.6 | 3 | 3 | | 3 |
| Q5CYQ0 | 40S ribosomal protein S27 (Fragment) | cgd7_1460 | 9.6092 | 13.298 | 24.4 | 2 | 4 | | 2 |
| Q5CUZ9 | 60S ribosomal protein L35 | cgd3_830 | 14.258 | 13.201 | 26.2 | 4 | 4 | | 4 |
| Q5CRH4 | Uncharacterized protein | cgd5_2760 | 33.463 | 13.185 | 17.9 | 5 | 5 | | 5 |
| Q5CQN9 | Emap RNA binding domain protein (N terminal low complexity region) | cgd4_3880 | 45.276 | 13.125 | 6 | 2 | 2 | | 2 |
| A3FQD5 | Vesicle transport protein, putative | cgd1_1890 | 26.039 | 13.086 | 12.8 | 3 | 3 | | 3 |
| Q7YYS4 | Uncharacterized protein | 1MB.494 | 65.819 | 12.888 | 9.4 | 5 | 6 | | 5 |
| A3FPL6 | cAMP-dependent protein kinase regulatory subunit (Fragment) | cgd7_120 | 38.794 | 12.838 | 13.6 | 6 | 7 | | 6 |
| Q5CRP3 | Extracellular protein | cgd5_2020 | 46.277 | 12.753 | 21.1 | 6 | 6 | | 6 |
| A3FPL4 | Uncharacterized protein | cgd7_20 | 24.121 | 12.734 | 20.8 | 4 | 5 | | 4 |
| Q86PR0 | TSP1 domain-containing protein TSP11 | --- | 129.23 | 12.698 | 4.3 | 4 | 4 | | 4 |
| F0X414 | Cgd8_360 protein | cgd8_360 | 26.388 | 12.685 | 24.8 | 5 | 5 | | 5 |
| F0X4U2 | 40S ribosomal protein SA | cgd3_2090 | 28.31 | 12.651 | 16 | 4 | 8 | | 4 |
| Q5CVQ4 | Endoplasmic reticulum oxidoreductin | cgd8_3190 | 45.729 | 12.634 | 7.8 | 4 | 4 | | 4 |
| Q7YYW1 | Cgd6_3270 protein | cgd6_3270 | 35.227 | 12.615 | 8.3 | 2 | 2 | | 2 |
| A3FQH2 | Eukaryotic translation initiation factor 3 subunit I | cgd5_680 | 29.63 | 12.612 | 21.1 | 5 | 5 | | 5 |
| Q5CTE4 | Uncharacterized protein | cgd2_3350 | 32.682 | 12.609 | 10.8 | 3 | 3 | | 3 |
| Q6YBX8 | Adenosylhomocysteinase | SAHH | 55.566 | 12.552 | 10.8 | 4 | 4 | | 4 |
| Q5CRI2 | Predicted mannitol dehydrogenase zinc dependent alcohol dehydrogenase like rossmann fold (Fragment) | cgd5_2670 | 40.057 | 12.523 | 11 | 3 | 3 | | 3 |
| Q208C2 | Acyl-CoA-binding protein | ACBP1 | 29.722 | 12.421 | 3.4 | 1 | 1 | | 1 |
| Q5CTX7 | Eukaryotic translation initiation factor 3 subunit L | cgd2_1160 | 77.539 | 12.36 | 4.9 | 2 | 2 | | 2 |
| F0X4P2 | Cgd8_3900 protein | cgd8_3900 | 48.602 | 12.306 | 11.7 | 5 | 6 | | 5 |
| Q7YYJ0 | BT1 family protein | 1MB.195 | 47.701 | 12.262 | 5.8 | 2 | 2 | | 2 |
| Q5CU09 | Uncharacterized protein (Fragment) | cgd2_760 | 38.834 | 12.127 | 4.4 | 1 | 1 | | 1 |
| Q5CY89 | Importin/karyopherin (Arm repeats) | cgd7_3270 | 127.56 | 12.09 | 2.1 | 2 | 2 | | 2 |
| Q5CQQ0 | Eukaryotic translation initiation factor 3 subunit C | cgd4_3770 | 116.32 | 12.07 | 5.9 | 5 | 5 | | 5 |
| Q5CQD4 | 26S proteasome regulatory subunit S4 like AAA ATpase | cgd4_1170 | 49.646 | 12.057 | 9.4 | 4 | 4 | | 4 |
| Q5CYU9 | Hypothetical transmembrane protein | cgd7_930 | 67.22 | 11.893 | 3.8 | 2 | 2 | | 2 |
| Q5CQL0 | Lipase/esterase, putative | cgd4_300 | 39.915 | 11.867 | 13.7 | 4 | 5 | | 4 |
| F0X4S3 | Cgd2_170 protein | cgd2_170 | 14.703 | 11.797 | 15.3 | 2 | 4 | | 2 |
| Q7YXZ4 | Uncharacterized protein | 1MB.114 | 32.042 | 11.774 | 11.7 | 3 | 7 | | 3 |
| Q7YYV5 | Ribosomal protein L32, probable | 1MB.429 | 16.14 | 11.683 | 34.3 | 5 | 8 | | 5 |
| F0X5F7 | Cgd8_2490 protein (Fragment) | cgd8_2490 | 58.464 | 11.475 | 9.1 | 4 | 5 | | 4 |
| Q5CUI5 | DNAj domain, possible transmembrane domain | cgd3_2690 | 72.943 | 11.459 | 6.2 | 4 | 4 | | 4 |
| Q5CTZ7 | Putative T complex chaperonin (Fragment) | cgd2_900 | 60.979 | 11.405 | 7.4 | 4 | 5 | | 4 |
| Q7YYY7 | Uncharacterized protein | 1MB.809 | 232.55 | 11.367 | 1.8 | 3 | 3 | | 3 |
| Q5CSF4 | DNAJ like chaperone, 4 or more transmembrane domains | cgd1_2970 | 44.213 | 11.357 | 7.7 | 3 | 3 | | 3 |
| Q5CVW6 | Sushi-domain containing secreted protein with a signal peptide, low complexity region followed by a sushi domain | cgd8_2530 | 113.39 | 11.321 | 3.3 | 4 | 4 | | 4 |
| Q5CYN0 | Threonyl-tRNA synthetase (RNA binding domain TGS+HxxxH+tRNA synthetase) | cgd7_1710 | 87.616 | 11.261 | 6 | 5 | 6 | | 5 |
| Q5CUP9 | Uncharacterized protein | cgd3_1940 | 19.982 | 11.176 | 30.8 | 5 | 7 | | 5 |
| Q5CSG8 | YyaF/YCHF TRANSFAC/OBG family small GTpase plus RNA binding domain TGS (Fragment) | cgd1_2760 | 44.678 | 11.143 | 8.5 | 3 | 3 | | 3 |
| Q7YY06 | Ribosomal protein S23 | 1MB.93 | 16.066 | 11.064 | 20.7 | 4 | 5 | | 4 |
| Q968X7 | Pyruvate dehydrogenase [NADP(+)] | PFOR | 217.55 | 11.001 | 1.7 | 4 | 4 | | 4 |
| Q5CUC2 | Uncharacterized protein | cgd3_3410 | 176.35 | 10.988 | 0.8 | 1 | 1 | | 1 |
| Q5CPD7 | Tryptophan synthase trpB of possible bacterial origin (Fragment) | cgd5_4560 | 49.421 | 10.923 | 4.5 | 2 | 2 | | 2 |
| Q5CXY0 | Signal peptide-containing protein with transmembrane domain | cgd7_4490 | 182.97 | 10.579 | 4.3 | 6 | 6 | | 6 |
| Q5CVM8 | 60S ribosomal protein L34 (Fragment) | cgd8_3480 | 15.748 | 10.551 | 21.7 | 3 | 7 | | 3 |
| Q5CPM7 | Uncharacterized protein | cgd2_250 | 14.371 | 10.495 | 37.7 | 4 | 5 | | 4 |
| Q5CVD2 | Uncharacterized protein | cgd8_4510 | 112.08 | 10.481 | 3.2 | 2 | 2 | | 2 |
| Q5CSY3 | 40S ribosomal protein S19 (Fragment) | cgd1_850 | 17.365 | 10.478 | 22.1 | 3 | 4 | | 3 |
| Q5CQJ0 | 26S proteasome regulatory subunit 5a with a vWA domain and two ubiquitin interacting motifs (UIM) | cgd4_530 | 41.493 | 10.454 | 4.2 | 1 | 1 | | 1 |
| Q5CPL7 | Eukaryotic translation initiation factor 3 subunit B | cgd2_360 | 84.046 | 10.434 | 5.5 | 3 | 3 | | 3 |
| Q5CW37 | Secreted protein, signal peptide | cgd8_1760 | 138.97 | 10.337 | 2.3 | 3 | 3 | | 3 |
| A3FQD8 | Rab2 GTPase, putative | cgd1_2060 | 21.088 | 10.332 | 30.8 | 5 | 5 | | 5 |
| Q5CYK7 | Membrane protein with multiple cysteines at the N-terminus | cgd7_1960 | 62.374 | 10.281 | 6 | 3 | 3 | | 3 |
| Q5CQI9 | V-type proton ATPase subunit C | cgd4_540 | 47.851 | 10.255 | 6.4 | 3 | 3 | | 3 |
| Q5CQI4 | Uncharacterized protein | cgd4_590 | 14.689 | 10.248 | 11.6 | 2 | 2 | | 2 |
| Q5CRH6 | Uncharacterized protein | cgd5_2740 | 59.827 | 10.222 | 2.3 | 1 | 1 | | 1 |
| A3FPZ9 | Uncharacterized protein (Fragment) | cgd8_4110 | 63.717 | 10.126 | 3.7 | 2 | 2 | | 2 |
| Q8MWQ1 | Oocyst wall protein (Fragment) | --- | 18.23 | 9.9228 | 34.9 | 3 | 20 | | 1 |
| Q5CR91 | Putative thioredoxin H-type of putative fungal or plant origin, small protein (Fragment) | cgd4_1650 | 11.9 | 9.8966 | 23.8 | 2 | 2 | | 2 |
| Q5CTW5 | Uncharacterized protein | cgd2_1290 | 49.119 | 9.8878 | 11.8 | 5 | 5 | | 5 |
| A3FPY2 | Uncharacterized protein | cgd8_1040 | 23.972 | 9.8706 | 10.2 | 2 | 2 | | 2 |
| Q5CY18 | Ribosomal protein L38 (Fragment) | cgd7_4050 | 9.5072 | 9.8064 | 36.6 | 4 | 10 | | 4 |
| Q5CUZ5 | Uncharacterized protein | cgd3_880 | 15.738 | 9.7919 | 16.2 | 2 | 2 | | 2 |
| Q5CRW0 | Conserved protein having 3 transmembrane domains and possible ER retention motif possible ER protein | cgd5_1270 | 43.315 | 9.7104 | 10.1 | 3 | 3 | | 3 |
| Q5CYL0 | CDC68 like aminopeptidase family chromatinic protein (Possible inactive enzyme) | cgd7_1930 | 128.07 | 9.6676 | 3.5 | 4 | 4 | | 4 |
| Q5CUR1 | Protein transport protein SEC23 (Fragment) | cgd3_1820 | 84.85 | 9.6342 | 9.2 | 6 | 6 | | 6 |
| Q7YZ05 | Cgd6_5080 protein | cgd6_5080 | 58.129 | 9.3813 | 10.7 | 5 | 6 | | 5 |
| Q5CYQ3 | Possible phosphodiesterase/alkaline phosphatase D, of possible plant or bacterial origin | cgd7_1430 | 53.027 | 9.3002 | 6.9 | 3 | 4 | | 3 |
| Q5CVL6 | Coiled coil protein (Fragment) | cgd8_3600 | 57.656 | 9.1074 | 11.4 | 5 | 5 | | 5 |
| A3FPY4 | ATP-dependent RNA helicase, putative | cgd8_1820 | 46.037 | 9.0153 | 9.6 | 4 | 4 | | 4 |
| Q5CTN0 | MA3 domain containing protein | cgd2_2350 | 65.022 | 9.01 | 5.6 | 3 | 3 | | 3 |
| Q5CV80 | Uncharacterized protein | cgd8_5090 | 35.798 | 9.0024 | 13.1 | 3 | 5 | | 3 |
| Q5CT08 | V-type proton ATPase proteolipid subunit | cgd1_520 | 16.873 | 9.0015 | 10.9 | 2 | 6 | | 2 |
| Q5CVP1 | Uncharacterized protein | cgd8_3330 | 16.328 | 8.8882 | 7 | 1 | 1 | | 1 |
| Q5CSJ1 | Signal recognition particle SPR68 | cgd1_2500 | 73.252 | 8.8199 | 4.9 | 3 | 3 | | 3 |
| Q5CYY4 | Uncharacterized protein | cgd7_550 | 45.067 | 8.8178 | 10 | 3 | 3 | | 3 |
| A3FQI7 | Dolichol phosphate mannose synthase, putative | cgd5_2040 | 27.598 | 8.6658 | 7.9 | 2 | 2 | | 2 |
| Q5CTW0 | 26S proteasome regulatory subunit, S6a like AAA ATpase (Fragment) | cgd2_1350 | 51.754 | 8.6646 | 15 | 6 | 6 | | 6 |
| Q5CSZ3 | Dynein heavy chain | cgd1_750 | 595.64 | 8.6555 | 1 | 5 | 5 | | 5 |
| Q7YYK5 | 26s proteasome ATPase subunit, probable | 1MB.625 | 48.216 | 8.6546 | 12 | 5 | 8 | | 4 |
| Q8I6I5 | Aminopeptidase (Fragment) | AP1 | 33.206 | 8.6538 | 8.6 | 2 | 2 | | 2 |
| Q5CTN1 | R1 like alpha-glucan water dikinase | cgd2_2340 | 192.83 | 8.6512 | 3 | 4 | 4 | | 4 |
| Q5CXS3 | A surface protein with 2 conserved cysteines | cgd7_5140 | 27.921 | 8.5394 | 13.2 | 3 | 6 | | 3 |
| Q86PQ4 | TSP1 domain-containing protein TSP6 | 1MB.246 | 40.177 | 8.3926 | 10.9 | 4 | 4 | | 4 |
| Q6S5I9 | Oocyst wall protein 5 | COWP5 | 71.105 | 8.3659 | 5.2 | 3 | 3 | | 3 |
| Q5CUP2 | Putative choline kinase (Fragment) | cgd3_2030 | 47.536 | 8.3192 | 9.1 | 4 | 4 | | 4 |
| Q7YY39 | Retinitis pigmentosa GTPase regulator, possible | 1MB.47 | 35.339 | 8.2921 | 3.5 | 1 | 1 | | 1 |
| Q7YY66 | Uncharacterized protein | 56k.18 | 70.924 | 8.262 | 5.8 | 4 | 4 | | 4 |
| Q5CRQ5 | T-complex protein 1 subunit delta (Fragment) | cgd5_1880 | 60.341 | 8.1677 | 9.1 | 4 | 5 | | 4 |
| Q5CRH2 | 40S ribosomal protein S10 | cgd5_2780 | 14.75 | 8.0911 | 25 | 3 | 3 | | 3 |
| Q5CPN1 | Glycerol-3-phosphate dehydrogenase [NAD(+)] | cgd2_210 | 46.077 | 8.0041 | 6.5 | 3 | 4 | | 3 |
| F0X537 | Uncharacterized protein | --- | 17.771 | 7.9819 | 6.6 | 1 | 1 | | 1 |
| Q5CV69 | Signal peptide, 4+ transmembrane domains | cgd8_5220 | 39.644 | 7.8299 | 9.9 | 3 | 3 | | 3 |
| Q5CU01 | Uncharacterized protein | cgd2_850 | 35.434 | 7.8296 | 11.5 | 4 | 5 | | 4 |
| Q7YY78 | Protease, possible | 56k.06 | 169.13 | 7.821 | 0.9 | 1 | 1 | | 1 |
| Q7YYF1 | Cgd6_2440 protein | cgd6_2440 | 30.697 | 7.797 | 3.4 | 1 | 2 | | 1 |
| Q7YYT0 | 26s proteasome non-ATPase regulatory subunit, probable | 1MB.479 | 48.797 | 7.7786 | 8.3 | 3 | 3 | | 3 |
| F0X4Y1 | Cgd4_1070 protein | cgd4_1070 | 27.186 | 7.7172 | 14.5 | 3 | 3 | | 3 |
| Q5CVH3 | Ribosomal protein S29 (Fragment) | cgd8_4050 | 7.6628 | 7.6764 | 26.6 | 2 | 2 | | 2 |
| Q5CVI1 | NAP, nucleosome assembly protein (Fragment) | cgd8_3970 | 23.649 | 7.6658 | 13.2 | 2 | 2 | | 2 |
| Q5CUL2 | Proliferation-associated protein 2G4 metalloprotease, creatinase/aminopeptidase fold | cgd3_2390 | 42.383 | 7.6015 | 5 | 2 | 2 | | 2 |
| Q5CTT4 | Dolichyl-diphosphooligosaccharide--protein glycosyltransferase 48 kDa subunit | cgd2_1650 | 53.572 | 7.5546 | 5.6 | 2 | 2 | | 2 |
| Q5CXW0 | Uncharacterized protein | cgd7_4720 | 26.151 | 7.5302 | 10.5 | 2 | 3 | | 2 |
| A3FQG1 | Aspartyl (Acid) protease, putative | cgd1_3690 | 88.41 | 7.4796 | 2.4 | 2 | 2 | | 2 |
| Q5CU29 | Proteasome subunit beta (Fragment) | cgd2_530 | 22.632 | 7.4642 | 14.2 | 3 | 4 | | 3 |
| Q5CSX6 | Uncharacterized protein | cgd1_950 | 54.123 | 7.4151 | 5.2 | 3 | 3 | | 3 |
| Q7YY02 | E2F-related protein, possible | 1MB.103 | 70.296 | 7.3873 | 4.1 | 2 | 2 | | 2 |
| Q5CS54 | Uncharacterized protein | cgd5_180 | 22.743 | 7.1694 | 13 | 2 | 2 | | 2 |
| Q5CQ04 | Uncharacterized protein | cgd8_110 | 14.758 | 7.0138 | 21.5 | 3 | 3 | | 3 |
| Q6TBR0 | UDP-N-acetyl-D-galactosamine:polypeptide N-acetylgalactosaminyltransferase T1 (Fragment) | --- | 72.52 | 6.9853 | 5 | 3 | 3 | | 3 |
| A3FQ08 | Palmitoyltransferase | cgd8_5050 | 22.161 | 6.9516 | 14.5 | 3 | 4 | | 3 |
| Q5CS11 | Musashi. RRM domain containing protein, splicing related (Fragment) | cgd5_700 | 53.217 | 6.936 | 6.5 | 2 | 2 | | 2 |
| Q5CYN1 | Histone H2B | cgd7_1700 | 14.362 | 6.9293 | 31.3 | 4 | 7 | | 3 |
| Q5CWJ3 | Serine protease, subtilase family, signal peptide | cgd6_4840 | 132.94 | 6.8855 | 2.1 | 2 | 2 | | 2 |
| Q7YYI9 | Oligosaccharyl transferase stt3 protein, probable | 1MB.197 | 81.897 | 6.8529 | 3 | 3 | 3 | | 3 |
| Q5CSR1 | 60S ribosomal protein L36 | cgd1_1660 | 12.025 | 6.8194 | 21.5 | 2 | 2 | | 2 |
| Q5CRU0 | Large cysteine rich protein | cgd5_1490 | 298.65 | 6.7548 | 1.6 | 4 | 4 | | 4 |
| Q5CWA1 | ER lumen protein-retaining receptor (Fragment) | cgd8_1110 | 26.213 | 6.7244 | 6.8 | 2 | 2 | | 2 |
| Q5CUV6 | Uncharacterized protein | cgd3_1330 | 55.433 | 6.6365 | 7.2 | 2 | 3 | | 2 |
| Q5CPW0 | Secrted alpha/beta hydrolase superfamiy protein | cgd8_600 | 47.576 | 6.622 | 7.2 | 3 | 3 | | 3 |
| Q5CW59 | Ubiquitin carboxyl terminal hydrolase domain that is fused to a MATH domain | cgd8_1530 | 181.32 | 6.5948 | 2.2 | 3 | 3 | | 3 |
| Q5CW01 | Proliferating cell nuclear antigen | cgd8_2150 | 29.209 | 6.569 | 17.6 | 3 | 3 | | 3 |
| A3FQ73 | Stress-induced protein sti1-like protein, putative | cgd2_1850 | 37.658 | 6.5158 | 10.1 | 3 | 4 | | 3 |
| Q5CYL5 | 60S ribosomal protein L44 (Fragment) | cgd7_1880 | 12.143 | 6.5056 | 10.6 | 3 | 3 | | 3 |
| Q5CTF3 | Phosphoglucomutase, tandemly duplicated protein | cgd2_3260 | 63.248 | 6.4584 | 9.3 | 4 | 5 | | 2 |
| Q7YY17 | Glycerol-3-phosphate acyltransferase, probable (Fragment) | 1Mx.01 | 61.578 | 6.4481 | 3.9 | 2 | 2 | | 2 |
| Q5CXU9 | Phosducin related thioredoxin fold protein | cgd7_4840 | 31.088 | 6.3618 | 16.8 | 4 | 4 | | 4 |
| Q5CTH4 | N-ethylmaleimide-sensitive factor (NSF1)-like AAA ATpase involved in vesicular transport | cgd2_3010 | 82.529 | 6.3347 | 3.1 | 2 | 3 | | 2 |
| A3FQ81 | Translation elongation factor EF-1, subunit alpha, putative | cgd2_2070 | 58.636 | 6.308 | 7.8 | 4 | 4 | | 4 |
| Q5CUM1 | Uncharacterized protein | cgd3_2260 | 15.547 | 6.3004 | 9.3 | 1 | 1 | | 1 |
| Q7YY47 | 26s protease regulatory subunit 8, probable | 1MB.33 | 44.612 | 6.2481 | 11.5 | 5 | 11 | | 3 |
| Q5CU27 | Uncharacterized protein (Fragment) | cgd2_550 | 27.733 | 6.1623 | 7.4 | 2 | 2 | | 2 |
| Q5CRD7 | Uncharacterized protein | cgd5_3210 | 148.95 | 6.1554 | 0.6 | 1 | 1 | | 1 |
| A3FQK7 | Preprocathepsin c, putative | cgd4_2110 | 71.984 | 6.1554 | 1.1 | 1 | 1 | | 1 |
| A3FQ24 | BIS(5'-nucleosyl)-tetraphosphatase (Diadenosine tetraphosphatase), putative (Fragment) | cgd3_1950 | 11.453 | 6.0835 | 23.2 | 2 | 2 | | 2 |
| Q5CXR9 | Eukaryotic translation initiation factor 3 subunit H | cgd7_5180 | 50.469 | 6.0581 | 4.2 | 2 | 2 | | 2 |
| Q5CVB0 | Dbp5p-like eIF4A-1-family RNA SFII helicase | cgd8_4750 | 58.633 | 6.0355 | 4.4 | 2 | 2 | | 2 |
| Q5CTY2 | O-acyltransferase | cgd2_1090 | 58.553 | 6.0318 | 7.9 | 3 | 3 | | 3 |
| Q5CSF0 | ER lumen protein retaining receptor 1 KDEL recpetor 1 | cgd1_3010 | 32.575 | 5.8884 | 2.8 | 1 | 2 | | 1 |
| Q5CUQ3 | Protein with 2 transmembrane domains near N-terminus, possible ArgA, N-acetylglutamate synthase and related acetyltransferase, camello or GNAT | cgd3_1900 | 33.306 | 5.6876 | 6.5 | 2 | 2 | | 2 |
| Q5CW35 | Uncharacterized protein | cgd8_1780 | 79.034 | 5.6474 | 7.4 | 3 | 3 | | 3 |
| Q5CTS3 | Mannose-1-phosphate guanylyltransferase | cgd2_1770 | 47.61 | 5.6409 | 9.4 | 3 | 3 | | 3 |
| Q7YYY6 | Uncharacterized protein | 1MB.813 | 104.52 | 5.5981 | 2.9 | 4 | 4 | | 4 |
| Q5CUE6 | Rab5 like small GTpase | cgd3_3150 | 24.206 | 5.5806 | 14.7 | 3 | 3 | | 3 |
| A3FQ55 | Dynein light chain | cgd2_740 | 10.265 | 5.5591 | 12.4 | 1 | 2 | | 1 |
| Q5CT27 | 40S ribosomal protein S21 (Fragment) | cgd1_300 | 8.7199 | 5.5584 | 18.8 | 2 | 2 | | 2 |
| Q5CYU8 | Splicing factor RRM domain containing protein T22E16.120 SC35-like splicing factor | cgd7_940 | 33.207 | 5.5349 | 4.5 | 1 | 1 | | 1 |
| Q7YY11 | Uncharacterized protein | 1Mx.08 | 141.45 | 5.4997 | 0.9 | 1 | 1 | | 1 |
| F0X501 | Cgd3_3930 protein | cgd3_3930 | 16.86 | 5.4878 | 18.8 | 3 | 5 | | 3 |
| Q5CTP8 | Signal peptide plus transmembrane domain or GPI anchor | cgd2_2160 | 112.13 | 5.4321 | 2.8 | 3 | 3 | | 3 |
| A3FPY0 | Glutamate--tRNA ligase (Fragment) | cgd8_790 | 63.393 | 5.411 | 3.6 | 2 | 2 | | 2 |
| Q5CTC4 | Uncharacterized protein | cgd2_3580 | 67.445 | 5.2897 | 2.5 | 1 | 1 | | 1 |
| Q5CVN0 | Methionyl-tRNA synthetase of possible bacterial origin (Fragment) | cgd8_3460 | 67.223 | 5.2833 | 3.4 | 2 | 2 | | 2 |
| A3FPY6 | Uncharacterized protein | cgd8_2120 | 59.363 | 5.2525 | 5.3 | 3 | 3 | | 3 |
| Q5CPQ9 | Uncharacterized protein | cgd4_4400 | 32.894 | 5.2403 | 5.9 | 2 | 2 | | 2 |
| Q5CQF3 | Uncharacterized protein | cgd4_940 | 31.902 | 5.1982 | 6.8 | 1 | 1 | | 1 |
| Q9Y0A8 | Adenylate kinase | AK | 24.228 | 5.1904 | 11.8 | 3 | 3 | | 3 |
| Q5CXL6 | Alanyl-tRNA synthetase (With HxxxH domain) (Fragment) | cgd6_560 | 114.04 | 5.1729 | 3.2 | 3 | 3 | | 3 |
| Q5CVB8 | Uncharacterized protein | cgd8_4660 | 45.395 | 5.123 | 6.8 | 2 | 2 | | 2 |
| Q5CQF6 | Uncharacterized protein | cgd4_910 | 64.74 | 5.1046 | 5.1 | 3 | 3 | | 3 |
| Q5CVR0 | Uncharacterized protein | cgd8_3120 | 30.506 | 5.0622 | 9.8 | 3 | 4 | | 3 |
| A3FQ00 | Uncharacterized protein | cgd8_4250 | 27.478 | 5.0609 | 11.2 | 2 | 2 | | 2 |
| Q5CRW6 | Uncharacterized protein | cgd5_1210 | 44.618 | 5.0432 | 5.9 | 2 | 2 | | 2 |
| Q5CY97 | T-complex protein 1 subunit epsilon (Fragment) | cgd7_3180 | 60.475 | 5.0422 | 5.2 | 4 | 4 | | 4 |
| Q5CYT8 | Apicomplexan conserved protein with 2 or more transmembrane domains | cgd7_1060 | 39.904 | 5.0379 | 5.5 | 2 | 2 | | 2 |
| Q27551 | Bifunctional dihydrofolate reductase-thymidylate synthase (Fragment) | DHFR-TS | 57.616 | 4.9867 | 5.4 | 3 | 3 | | 3 |
| Q5CSW6 | Uncharacterized protein | cgd1_1060 | 122.04 | 4.9819 | 2.9 | 3 | 3 | | 3 |
| Q5CV19 | Uncharacterized protein | cgd3_600 | 160.63 | 4.9701 | 3.4 | 4 | 4 | | 4 |
| Q5CRE8 | Carboxylic ester hydrolase | cgd5_3070 | 65.363 | 4.9491 | 4.1 | 2 | 2 | | 2 |
| Q5CVR2 | Valyl-tRNA synthetase | cgd8_3100 | 120.26 | 4.9353 | 2.8 | 3 | 3 | | 3 |
| Q5CRB3 | Uncharacterized protein | cgd5_3490 | 32.514 | 4.926 | 14.8 | 4 | 4 | | 4 |
| Q7YYA0 | Tyrosyl-tRNA synthetase, probable | 1MB.360 | 42.939 | 4.9195 | 7.7 | 2 | 2 | | 2 |
| Q5CPT1 | Uncharacterized protein | cgd4_4170 | 17.806 | 4.8788 | 4.6 | 1 | 1 | | 1 |
| Q5CUS8 | Uncharacterized protein (Fragment) | cgd3_1630 | 21.432 | 4.8252 | 13.4 | 2 | 2 | | 2 |
| Q5CWA8 | Glycyl-tRNA synthetase | cgd8_1030 | 74.948 | 4.8167 | 3.4 | 2 | 2 | | 2 |
| Q5CR13 | Uncharacterized protein | cgd4_2510 | 43.21 | 4.8132 | 3.1 | 1 | 2 | | 1 |
| Q5CSU4 | Apicomplexa specific secreted protein Pf (23508265), signal peptide | cgd1_1290 | 29.545 | 4.7942 | 8.6 | 2 | 2 | | 2 |
| Q5CW91 | Mitochondrial ADP/ATP-transporter, integral membrane protein with 4 transmembrane domains (Fragment) | cgd8_1210 | 36.984 | 4.7909 | 5.8 | 2 | 2 | | 2 |
| Q5CYZ1 | Uncharacterized protein | cgd7_490 | 24.32 | 4.7747 | 12.6 | 3 | 3 | | 3 |
| A3FQQ3 | Uncharacterized protein (Fragment) | cgd4_4030 | 22.456 | 4.757 | 12.4 | 2 | 2 | | 2 |
| Q5CW05 | RAN binding domain | cgd8_2100 | 45.779 | 4.7385 | 3.7 | 1 | 1 | | 1 |
| Q5CVH7 | Histidyl-tRNA synthetase | cgd8_4010 | 110.13 | 4.6946 | 0.8 | 1 | 1 | | 1 |
| Q5CR24 | 60S ribosomal protein L31 | cgd4_2400 | 13.093 | 4.6571 | 13.9 | 2 | 4 | | 2 |
| Q5CQR3 | Uncharacterized protein | cgd4_3630 | 95.558 | 4.643 | 0.7 | 1 | 1 | | 1 |
| Q5CS90 | Uncharacterized protein | cgd1_3650 | 40.966 | 4.6245 | 2.3 | 1 | 1 | | 1 |
| Q5CT72 | Uncharacterized protein | cgd2_4180 | 16.268 | 4.62 | 7.1 | 1 | 1 | | 1 |
| Q7YYD4 | Cgd6_2620 protein | cgd6_2620 | 27.463 | 4.5936 | 8 | 1 | 1 | | 1 |
| Q5CTE2 | Proteasome regulatory subunit Rpn12 family (Fragment) | cgd2_3370 | 32.765 | 4.5702 | 6.4 | 2 | 2 | | 2 |
| Q7YYQ5 | Rab1a protein, probable | 1MB.524 | 23.938 | 4.5464 | 13.1 | 3 | 4 | | 3 |
| Q5CVA6 | Proteolipid subunit of the vacuolar ATpase | cgd8_4790 | 19.354 | 4.5454 | 12.2 | 2 | 3 | | 2 |
| Q5CU74 | PUG domain fused to an UBA domain | cgd3_3950 | 35.004 | 4.424 | 8.7 | 2 | 2 | | 2 |
| Q5CXS9 | Uncharacterized protein | cgd7_5080 | 76.915 | 4.4089 | 3.7 | 3 | 3 | | 3 |
| Q7YYH0 | Uncharacterized protein | 1MB.232 | 38.318 | 4.4057 | 5.8 | 2 | 3 | | 2 |
| Q5CQL8 | Ubc1p like ubiquitin-conjugating enzyme E2 fused to a UBA domain (UBC+UBA) (Fragment) | cgd4_210 | 22.782 | 4.3141 | 10.2 | 2 | 2 | | 2 |
| Q5CPP5 | SIK1 nucleolar protein Nop56 | cgd2_50 | 56.698 | 4.2788 | 3 | 1 | 1 | | 1 |
| Q5CVP3 | Ylr022cp-like protein that has a C2H2 zinc finger and is a component of the exosome (Fragment) | cgd8_3310 | 44.014 | 4.2545 | 2.1 | 1 | 1 | | 1 |
| Q5CWA0 | HBS1 eRFS. GTpase. (RNA metabolism translation) | cgd8_1120 | 59.839 | 4.2537 | 2.8 | 1 | 1 | | 1 |
| U3L2T5 | RNA binding protein (Fragment) | KhRNAb | 22.757 | 4.1473 | 4.4 | 1 | 1 | | 1 |
| Q5CTU7 | Possible translation initiation factor with possible PINT domain | cgd2_1500 | 62.963 | 4.1371 | 3.5 | 2 | 2 | | 2 |
| F0X4F0 | Cgd7_5270 protein | cgd7_5270 | 38.131 | 4.1119 | 7.8 | 3 | 3 | | 3 |
| Q5CWD6 | Possible apicomplexan protein (Fragment) | cgd8_730 | 19.898 | 4.095 | 5.4 | 1 | 2 | | 1 |
| Q5CSN3 | Uncharacterized protein | cgd1_1970 | 20.728 | 4.093 | 13.8 | 2 | 2 | | 2 |
| Q5CUS4 | Uncharacterized protein | cgd3_1680 | 24.334 | 4.0669 | 4.3 | 1 | 1 | | 1 |
| Q5CU22 | Uncharacterized protein | cgd2_610 | 29.374 | 4.0302 | 6.1 | 2 | 2 | | 2 |
| F0X3I3 | V-type proton ATPase subunit | cgd5_3340 | 45.702 | 4.0241 | 2.3 | 1 | 1 | | 1 |
| Q5CPR9 | Beta adaptin (Fragment) | cgd4_4300 | 87.313 | 3.9527 | 3.1 | 2 | 2 | | 2 |
| Q7YYP5 | Hydroxyproline-rich glycoprotein dz-hrgp, probable | 1MB.549 | 21.311 | 3.9257 | 6.4 | 1 | 1 | | 1 |
| Q5CY17 | Signal recognition particle subunit SRP72 | cgd7_4060 | 77.642 | 3.9073 | 2.2 | 1 | 1 | | 1 |
| A3FQL4 | Uncharacterized protein | cgd4_2710 | 8.887 | 3.9041 | 22.1 | 2 | 2 | | 2 |
| Q7YYG6 | DEAD/DEAH box helicase | 1MB.237 | 84.451 | 3.9039 | 1.8 | 1 | 1 | | 1 |
| Q5CR18 | Uncharacterized protein | cgd4_2460 | 33.797 | 3.8777 | 7.4 | 2 | 2 | | 2 |
| Q5CT63 | Secreted insulinase-like peptidase (Fragment) | cgd2_4270 | 141.06 | 3.848 | 1.9 | 2 | 2 | | 2 |
| Q5CRU1 | Cysteine rich protein having a signal peptide | cgd5_1480 | 140.41 | 3.8056 | 1.9 | 3 | 3 | | 3 |
| Q5CZ05 | Transmembrane 9 superfamily member (Fragment) | cgd7_330 | 71.948 | 3.8036 | 1.6 | 1 | 1 | | 1 |
| D7F4K5 | Gp40/15 glycoprotein (Fragment) | --- | 30.733 | 3.8036 | 48.1 | 13 | 47 | | 0 |
| F0X4S9 | Serine/threonine-protein phosphatase | cgd7_2670 | 30.084 | 3.7964 | 5.4 | 1 | 1 | | 1 |
| Q5CT33 | ALG-2 like alpha-1,3 mannosyltransferase (Fragment) | cgd1_230 | 54.653 | 3.7941 | 3.8 | 2 | 2 | | 2 |
| A3FQ79 | Mitogen-activated protein kinase | cgd2_1960 | 80.115 | 3.7885 | 1.8 | 1 | 1 | | 1 |
| Q5CT05 | Predicted secreted protein, signal peptide | cgd1_570 | 27.509 | 3.7631 | 9 | 2 | 3 | | 2 |
| Q5CRB6 | Nucleotidase (5'-nucleotidase/2'-cyclic phosphodiesterase) of the calcineurin superfamily | cgd5_3460 | 80.347 | 3.7026 | 4.4 | 3 | 3 | | 3 |
| O96498 | UMP kinase | UMPK | 22.795 | 3.6699 | 10.1 | 2 | 2 | | 2 |
| Q5CW84 | Centrin, caltractin (Fragment) | cgd8_1280 | 18.856 | 3.6358 | 9.6 | 1 | 1 | | 1 |
| Q5CVV2 | Secreted glucose methanol choline like oxidoreductase of the FAD dependent oxidoreductase like fold, signal peptide | cgd8_2670 | 76.892 | 3.584 | 2.3 | 2 | 2 | | 2 |
| Q5CRV5 | SPAC25G10.01-like RRM domain containing protein | cgd5_1330 | 29.614 | 3.5837 | 11.5 | 3 | 3 | | 3 |
| A3FQ17 | Rhomboid-like protein (Fragment) | cgd3_980 | 23.237 | 3.5348 | 8.6 | 2 | 2 | | 2 |
| Q5CXM1 | Synaptobrevin/VAMP-like protein | cgd6_510 | 26.128 | 3.4986 | 8 | 2 | 2 | | 2 |
| Q5CWL0 | 5kows transcription initiation protein SPT5 (Fragment) | cgd6_4650 | 97.299 | 3.4983 | 1.1 | 1 | 1 | | 1 |
| Q7YY43 | Cgd6_960 protein | cgd6_960 | 66.26 | 3.4933 | 4.1 | 2 | 2 | | 2 |
| Q7YYZ9 | Lectin-domain protein | 1MB.779 | 52.431 | 3.4883 | 3 | 1 | 1 | | 1 |
| D6P6P1 | Cgd6_200-like protein (Fragment) | --- | 15.314 | 3.4804 | 91.4 | 10 | 191 | | 1 |
| Q5CSG3 | Syntaxin 5A ortholog, possible transmembrane domain or GPI at C-terminus (Fragment) | cgd1_2830 | 37.824 | 3.4737 | 4.3 | 1 | 1 | | 1 |
| Q7YZ48 | Uncharacterized protein | 1MB.690 | 65.508 | 3.4384 | 2.3 | 1 | 1 | | 1 |
| Q5CRH8 | Large protein containing a signal peptide | cgd5_2720 | 224.01 | 3.4193 | 1 | 2 | 2 | | 2 |
| Q5CR47 | TBC domain containing protein | cgd4_2130 | 39.781 | 3.404 | 5.8 | 2 | 2 | | 2 |
| Q5CSP1 | Uncharacterized protein | cgd1_1870 | 54.574 | 3.3484 | 6.4 | 2 | 3 | | 2 |
| A3FQQ1 | Ubiquitin-conjugating enzyme E2, putative (Fragment) | cgd8_580 | 15.783 | 3.3429 | 20 | 2 | 2 | | 2 |
| Q5CT15 | Possible emp24/gp25L/p24 family protein, transmembrane domain (Fragment) | cgd1_430 | 14.508 | 3.3233 | 12.3 | 1 | 1 | | 1 |
| Q5CYT6 | eIF3-p47 with JAB/PAD domain | cgd7_1080 | 38.972 | 3.3144 | 2.3 | 1 | 1 | | 1 |
| Q7YYR7 | 40S ribosomal protein S30 | 1MB.506 | 6.5776 | 3.2706 | 13.8 | 1 | 1 | | 1 |
| Q5CTV9 | Hypothetical coiled coil protein | cgd2_1370 | 98.151 | 3.2657 | 2.3 | 1 | 1 | | 1 |
| Q5CTJ0 | Uncharacterized protein | cgd2_2790 | 38.814 | 3.2232 | 2.4 | 1 | 1 | | 1 |
| Q5CPP7 | Cysteine desulfurase/selenocysteine lyase-like PLP dependent transferase superfamily protein | cgd2_30 | 56.626 | 3.1746 | 3.8 | 1 | 1 | | 1 |
| Q5CUW8 | Possible prefoldin-related protein (Fragment) | cgd3_1200 | 14.718 | 3.1379 | 17.3 | 2 | 3 | | 2 |
| Q5CVI3 | eIF-2B gamma, eukaryotic translation initiation factor 2B subunit 3 that has a nucleotide diphospho sugar transferase at the N-terminus and a UDP N-acetylglucosamine acyltransferase at the C-terminus | cgd8_3940 | 56.207 | 3.119 | 2.2 | 1 | 1 | | 1 |
| Q5CS92 | Uncharacterized protein | cgd1_3630 | 80.35 | 3.1012 | 2.1 | 1 | 1 | | 1 |
| Q7YYB2 | Sucrose-phosphatase, possible | 1MB.336 | 37.239 | 3.0794 | 9 | 3 | 3 | | 3 |
| Q5CQ82 | SNF7 ortholog | cgd5_3790 | 23.195 | 3.0782 | 6.3 | 1 | 1 | | 1 |
| Q7YYX7 | Cgd6_5430 protein | cgd6_5430 | 48.225 | 3.0237 | 4 | 1 | 1 | | 1 |
| Q5CVL7 | Predicted secreted protein | cgd8_3590 | 60.931 | 2.9901 | 3 | 2 | 2 | | 2 |
| Q5CUR3 | Uncharacterized protein | cgd3_1790 | 127.43 | 2.9624 | 1 | 1 | 1 | | 1 |
| Q5CS13 | RuvB-like helicase | cgd5_670 | 53.632 | 2.9403 | 4 | 2 | 2 | | 2 |
| Q5CXS7 | Protein disulfide isomerase, signal peptide plus ER retention motif | cgd7_5100 | 64.531 | 2.9149 | 4.7 | 2 | 2 | | 2 |
| Q5CTR2 | Apicomplexan protein with signal peptide and 3 transmembrane domains | cgd2_2000 | 20.812 | 2.9085 | 4.8 | 1 | 2 | | 1 |
| Q7YZ56 | Ubiquitinyl hydrolase 1 | 1MB.672 | 104.51 | 2.905 | 1.3 | 1 | 1 | | 1 |
| Q5CYD8 | ABC transporter with ATpase domain plus 3 transmembrane regions | cgd7_2730 | 80.775 | 2.9003 | 2.3 | 2 | 2 | | 2 |
| Q5CRY0 | Uncharacterized protein (Fragment) | cgd5_1050 | 47.86 | 2.8891 | 2.2 | 1 | 1 | | 1 |
| Q5CYJ8 | Hch1p like mystery proteins (Fragment) | cgd7_2050 | 44.95 | 2.8611 | 4.4 | 1 | 1 | | 1 |
| Q5CUR9 | Uncharacterized protein | cgd3_1730 | 32.978 | 2.856 | 5 | 1 | 1 | | 1 |
| Q5CUE5 | Uncharacterized protein | cgd3_3160 | 36.633 | 2.8488 | 2.9 | 1 | 1 | | 1 |
| Q5CUU3 | GAF domain containing protein | cgd3_1460 | 61.159 | 2.8285 | 1.9 | 1 | 1 | | 1 |
| Q5CQ34 | Uncharacterized protein | cgd5_4300 | 80.83 | 2.8153 | 2.2 | 2 | 2 | | 2 |
| Q5CPG0 | Zfwd1 protein, CCCH like RNA binding domain fused to WD repeats | cgd3_200 | 44.04 | 2.7587 | 2.7 | 1 | 1 | | 1 |
| Q5CR29 | Multitransmembrane protein with signal peptide and GMGPP repeat at C-terminus | cgd4_2350 | 36.897 | 2.7508 | 3.4 | 1 | 1 | | 1 |
| Q5CTI3 | Putative biotin-(Acetyl-CoA carboxylase) ligase | cgd2_2890 | 33.83 | 2.7424 | 3.4 | 1 | 1 | | 1 |
| A3FQ29 | cGMP phosphodiesterase A4 | cgd3_2320 | 112.68 | 2.7143 | 0.7 | 1 | 1 | | 1 |
| Q5CS83 | Glucosamine-fructose-6-phosphate aminotransferase | cgd1_3730 | 76.468 | 2.7037 | 2.9 | 2 | 2 | | 2 |
| F0X4J2 | Cgd7_4730 protein | cgd7_4730 | 37.593 | 2.6981 | 2.3 | 1 | 1 | | 1 |
| A3FQB5 | Signal peptidase I | cgd1_440 | 16.256 | 2.6922 | 6.2 | 1 | 1 | | 1 |
| Q5CVS1 | SYG1/ ERD1 like integral membrane protein required for retention of ER lumen proteins, with 8-10 transmembrane domains | cgd8_3000 | 78.86 | 2.6288 | 2.1 | 2 | 2 | | 2 |
| Q5CSX8 | U2 snrnp-specific A' protein | cgd1_930 | 34.371 | 2.6258 | 4 | 1 | 1 | | 1 |
| Q5CSY6 | Palmitoyltransferase | cgd1_820 | 89.173 | 2.5944 | 1.4 | 1 | 1 | | 1 |
| Q7YZ65 | Uncharacterized protein | 1MB.644 | 47.773 | 2.5914 | 2.6 | 1 | 2 | | 1 |
| Q7YYX1 | Sel-1 protein, possible | 1MB.406 | 94.588 | 2.58 | 1.2 | 1 | 1 | | 1 |
| Q5CVM0 | Predicted secreted protein | cgd8_3560 | 57.682 | 2.5235 | 4.6 | 2 | 2 | | 2 |
| Q5CW00 | Shares a domain with poly(ADP) ribose glycohydrolases, some protein kinase A anchoring proteins and baculovirus HzNV Orf103, possible transmembrane domain within N-terminus | cgd8_2160 | 49.842 | 2.4927 | 2.7 | 1 | 1 | | 1 |
| A0A2H4U2H6 | COWP protein (Fragment) | COWP | 16.812 | 2.4819 | 54.1 | 6 | 54 | | 1 |
| Q5CWU1 | Large extracellular protein with a signal peptide, apple domain and a transmembrane region | cgd6_3730 | 252.7 | 2.4783 | 0.7 | 1 | 1 | | 1 |
| Q5CTU0 | Putative vacuolar protein sorting/targeting protein 26 | cgd2_1570 | 34.906 | 2.4736 | 3.9 | 1 | 1 | | 1 |
| Q5CYM7 | Phospholipid-transporting ATPase (Fragment) | cgd7_1760 | 143.92 | 2.4696 | 0.5 | 1 | 1 | | 1 |
| Q5CYB9 | Coatomer subunit delta | cgd7_2940 | 60.71 | 2.4668 | 2.2 | 1 | 1 | | 1 |
| Q5CPX0 | Histone deacetylase | cgd8_480 | 107.86 | 2.4668 | 1.2 | 1 | 1 | | 1 |
| Q5CV14 | Uncharacterized protein | cgd3_650 | 99.918 | 2.4476 | 1.7 | 2 | 2 | | 2 |
| Q5CR48 | PX domain containing protein | cgd4_2120 | 56.993 | 2.4424 | 2.4 | 1 | 1 | | 1 |
| Q5CPM2 | Kelch repeat-containing proteins that is fused to a HSP90-like ATpase | cgd2_300 | 141.2 | 2.4417 | 1 | 1 | 1 | | 1 |
| Q5CT18 | Uncharacterized protein (Fragment) | cgd1_390 | 33.611 | 2.4178 | 3.5 | 1 | 1 | | 1 |
| Q5CVZ6 | BRG1/brm-associated factor 53A like actin | cgd8_2200 | 46.26 | 2.3989 | 2.4 | 1 | 1 | | 1 |
| Q5CTZ6 | Peptidase'insulinase-like peptidase (Fragment) | cgd2_920 | 118.7 | 2.3845 | 1.9 | 2 | 2 | | 2 |
| Q5CV85 | Starch binding domain containing protein, possible plant origin | cgd8_5020 | 18.316 | 2.3705 | 5.7 | 1 | 1 | | 1 |
| Q5CS17 | Protein with 4 ankyrin repeats plus a bromodomain | cgd5_630 | 48.686 | 2.3582 | 2.3 | 1 | 1 | | 1 |
| A3FQ77 | dTDP-glucose 4-6-dehydratase-like protein, putative | cgd2_1900 | 38.11 | 2.3513 | 2.4 | 1 | 1 | | 1 |
| Q6S4W6 | Thymidine kinase | TK | 22.331 | 2.345 | 6.7 | 2 | 2 | | 2 |
| Q5CYV4 | eIF-3A like translation initiation factor that has a WD40 repeat-containing beta propeller | cgd7_880 | 65.402 | 2.3333 | 2.2 | 1 | 1 | | 1 |
| Q5CVF4 | Uncharacterized protein | cgd8_4260 | 13.384 | 2.3303 | 8.2 | 1 | 1 | | 1 |
| Q5CU73 | Yer007c-ap/MCT-1 like PUA RNA binding domain containing protein | cgd3_3970 | 20.183 | 2.3174 | 7.3 | 1 | 1 | | 1 |
| Q5CTA5 | Hypothetical small protein with 2 EF-hand domains, possible | cgd2_3790 | 12.126 | 2.301 | 9.6 | 1 | 1 | | 1 |
| Q5CR56 | Uncharacterized protein | cgd4_2020 | 76.368 | 2.2995 | 2.7 | 2 | 2 | | 2 |
| F0X5Q9 | Cgd1_3340 protein | cgd1_3340 | 33.872 | 2.2985 | 3 | 1 | 1 | | 1 |
| Q5CYV0 | Protein with 2 possible TPR domains, possible n-terminal acetyltransferase (Fragment) | cgd7_920 | 125.07 | 2.2673 | 0.7 | 1 | 1 | | 1 |
| Q5CPY4 | Uncharacterized protein | cgd8_320 | 61.519 | 2.2647 | 2.9 | 2 | 2 | | 2 |
| Q5CU99 | Uncharacterized protein (Fragment) | cgd3_3660 | 10.374 | 2.2645 | 12.1 | 1 | 1 | | 1 |
| A3FPT5 | Uncharacterized protein (Fragment) | cgd6_1650 | 21.918 | 2.2615 | 5.8 | 1 | 1 | | 1 |
| Q5CT28 | Ub6p like ubiquitin at N-terminus and ubiquitin C terminal hydrolase at the C-terminus (Fragment) | cgd1_290 | 56.679 | 2.2602 | 4.6 | 2 | 2 | | 2 |
| Q5CT81 | Uncharacterized protein | cgd2_4060 | 86.521 | 2.2524 | 1.5 | 1 | 1 | | 1 |
| Q5CQM1 | Uncharacterized protein | cgd4_180 | 172.23 | 2.2434 | 0.9 | 1 | 1 | | 1 |
| Q5CVR3 | Proteasome activator p28/ Ki autoantigen | cgd8_3090 | 27.11 | 2.2427 | 3 | 1 | 1 | | 1 |
| Q9NAZ9 | Ribonucleotide reductase R2 subunit | rnrR2 | 41.203 | 2.238 | 2.3 | 1 | 1 | | 1 |
| F0X4F7 | Cgd2_980 protein | cgd2_980 | 24.009 | 2.2201 | 4.9 | 1 | 1 | | 1 |
| Q5CTH1 | Uncharacterized protein | cgd2_3040 | 18.047 | 2.1975 | 4.9 | 1 | 2 | | 1 |
| Q5CQF9 | Uncharacterized protein | cgd4_870 | 118.39 | 2.1944 | 0.9 | 1 | 1 | | 1 |
| Q5CXY7 | Secreted acid phosphatase (Calcineurin family),signal peptide | cgd7_4420 | 93.749 | 2.1792 | 1.1 | 1 | 1 | | 1 |
| Q5CY04 | TCP-1 chaperonin | cgd7_4220 | 58.347 | 2.1768 | 4.9 | 2 | 2 | | 2 |
| Q5CS77 | Uncharacterized protein | cgd1_3790 | 145.09 | 2.1726 | 1 | 1 | 1 | | 1 |
| Q5CX78 | Fun12p GTpase translation initiation factor IF2 (Fragment) | cgd6_2180 | 100.88 | 2.1696 | 1.2 | 1 | 1 | | 1 |
| Q5CT25 | Oxysterol binding protein (Fragment) | cgd1_320 | 67.688 | 2.1628 | 2 | 1 | 1 | | 1 |
| Q5CVE8 | Fibrillarin RNA methylase | cgd8_4330 | 31.818 | 2.1613 | 3.7 | 1 | 1 | | 1 |
| A3FQN0 | Uncharacterized protein | cgd4_240 | 49.226 | 2.1357 | 4.6 | 2 | 2 | | 2 |
| Q5CV28 | Putative fucose translocatorw ith 8 transmembrane domains, within locus of 3 paralogous genes | cgd3_510 | 42.517 | 2.1339 | 3.9 | 2 | 3 | | 2 |
| Q5CSV0 | Predicted secreted protein, signal peptide | cgd1_1230 | 123.69 | 2.1275 | 1 | 1 | 1 | | 1 |
| Q5CXT5 | Coatomer complex beta (Fragment) | cgd7_5010 | 131.94 | 2.1081 | 1.6 | 2 | 2 | | 2 |
| Q5CS28 | Uncharacterized protein | cgd5_480 | 28.102 | 2.106 | 3.8 | 1 | 1 | | 1 |
| Q5CUU1 | Pp2c protein phosphatase 2 C, possible transmembrane domain near N-terminus | cgd3_1480 | 69.488 | 2.0818 | 2.6 | 2 | 2 | | 2 |
| Q7YYD1 | Cgd6_2650 protein | cgd6_2650 | 30.795 | 2.0683 | 4.4 | 1 | 1 | | 1 |
| Q7YYU4 | Uncharacterized protein | 1MB.453 | 83.392 | 2.0233 | 2.9 | 1 | 1 | | 1 |
| Q7YYE6 | E3 ubiquitin-protein ligase, probable | 1MB.271 | 90.001 | 2.0024 | 2 | 1 | 1 | | 1 |
| A3FQ91 | Phosphoprotein phosphatase related, putative | cgd2_2960 | 59.588 | 2.0024 | 3 | 1 | 1 | | 1 |
| Q5CRK6 | Uncharacterized protein (Fragment) | cgd5_2430 | 18.442 | 2.0008 | 6.1 | 1 | 1 | | 1 |
| Q5CUT3 | Alpha glucosidase-like faimly 31 glycosyl hydrolases | cgd3_1580 | 161.23 | 1.9966 | 0.5 | 1 | 1 | | 1 |
| Q5CRR6 | H/ACA ribonucleoprotein complex subunit | cgd5_1760 | 19.526 | 1.9851 | 4.9 | 1 | 1 | | 1 |
| Q8WQX4 | NifS-like protein | --- | 48.7 | 1.979 | 2.3 | 1 | 1 | | 1 |
| Q5CYA9 | Membrane protein conserved in eukaryotes | cgd7_3040 | 190.74 | 1.9763 | 1.1 | 2 | 2 | | 2 |
| Q7JMU6 | Cryptosporidium oocyst wall protein of 190,000 dalton (Fragment) | --- | 85.562 | 1.9668 | 66.7 | 41 | 296 | | 1 |
| Q5CQJ6 | Very large low complexity protein | cgd4_450 | 285.87 | 1.9487 | 0.8 | 1 | 1 | | 1 |
| Q5CTF7 | Uncharacterized protein | cgd2_3210 | 34.815 | 1.9248 | 3.7 | 1 | 1 | | 1 |
| Q5CYC2 | Abd1p RNA (Guanine-7-methyltransferase (Cap methyltransferase)) | cgd7_2910 | 77.578 | 1.9222 | 1.5 | 1 | 1 | | 1 |
| Q5CRM5 | Membrane associated thioredoxin | cgd5_2230 | 42.097 | 1.9104 | 6.1 | 2 | 2 | | 2 |
| Q5CVP7 | Importin alpha subunit (Fragment) | cgd8_3260 | 61.552 | 1.8954 | 2 | 1 | 1 | | 1 |
| Q5CRN0 | Large low complexity protein with proline/alanine-rich repeat | cgd5_2180 | 162.27 | 1.8934 | 0.9 | 1 | 1 | | 1 |
| Q5CQX7 | Polyketide synthase | cgd4_2900 | 1516.3 | 1.8874 | 0.1 | 2 | 2 | | 2 |
| Q5CTU8 | Uncharacterized protein | cgd2_1490 | 38.02 | 1.8759 | 3 | 1 | 2 | | 1 |
| A3FPP0 | Skp1 family protein, putative | cgd7_2500 | 18.528 | 1.8648 | 5.6 | 1 | 1 | | 1 |
| Q5CY91 | Membrane protein with no close homologs | cgd7_3250 | 29.859 | 1.86 | 3.4 | 1 | 1 | | 1 |
| Q5CRX2 | Cinnamyl-alcohol dehydrogenase-like nucleoside diphosphate sugar epimerase | cgd5_1140 | 50.323 | 1.8562 | 2.3 | 1 | 1 | | 1 |
| Q5CTX6 | Zinc finger protein | cgd2_1170 | 53.244 | 1.8296 | 4.2 | 2 | 2 | | 2 |
| Q5CVJ4 | Uncharacterized protein | cgd8_3830 | 14.503 | 1.8134 | 17.2 | 1 | 1 | | 1 |
| A3FQH4 | Uncharacterized protein | cgd5_780 | 142.78 | 1.7916 | 0.9 | 1 | 3 | | 1 |
| Q5CT07 | V-type proton ATPase proteolipid subunit (Fragment) | cgd1_540 | 16.986 | 1.7822 | 10.8 | 1 | 1 | | 1 |
| Q5CVS0 | Structural maintenance of chromosomes protein 6 | cgd8_3010 | 148.56 | 1.7633 | 0.6 | 1 | 2 | | 1 |
| Q5CSW4 | Alpha beta hydrolase (Fragment) | cgd1_1080 | 25.075 | 1.7608 | 5.5 | 1 | 1 | | 1 |
| Q5CV00 | Uncharacterized protein | cgd3_820 | 15.458 | 1.7511 | 7.7 | 1 | 1 | | 1 |
| Q5CY35 | Uncharacterized protein | cgd7_3830 | 68.992 | 1.7344 | 4.3 | 2 | 2 | | 2 |
| Q5CVS5 | Replication factor C like AAA ATpase (Fragment) | cgd8_2940 | 37.401 | 1.7315 | 3 | 1 | 1 | | 1 |
| Q5CQG8 | DNA topoisomerase 2 | cgd4_780 | 204.1 | 1.7302 | 0.6 | 1 | 1 | | 1 |
| Q5CY77 | Secreted alpha beta hydrolyse, signal peptide, secreted patatin like esterase (Ralstonia best hits) | cgd7_3390 | 90.39 | 1.7166 | 1 | 1 | 2 | | 1 |
| Q7YYZ0 | Uncharacterized protein | 1MB.802 | 18.569 | 1.7002 | 4.4 | 1 | 1 | | 1 |
| Q5CTZ9 | Drosophila CG6013 like HMG domain containing protein with a coiled coil region at the N-terminus | cgd2_870 | 26.326 | 1.6977 | 7 | 1 | 1 | | 1 |
| Q5CQU2 | Phosphoacetylglucosamine mutase | cgd4_3310 | 73.608 | 1.6916 | 2 | 1 | 1 | | 1 |
| Q5CSS4 | Rpn5 like 26S proteasomal regulatory subunit 12, PINT domain containing protein | cgd1_1530 | 65.613 | 1.6891 | 3 | 2 | 2 | | 2 |
| Q7YYS8 | Uncharacterized protein | 1MB.483 | 42.957 | 1.6869 | 1.8 | 1 | 1 | | 1 |
| Q5CUU7 | Protein kish (Fragment) | cgd3_1420 | 8.4012 | 1.6642 | 11.8 | 1 | 1 | | 1 |
| Q5CQW9 | RhoGAP domain containing protein with a Sec14D domain at the N-terminus (Fragment) | cgd4_2980 | 50.135 | 1.6619 | 3 | 1 | 1 | | 1 |
| Q5CQS4 | Uncharacterized protein | cgd4_3520 | 113.08 | 1.6218 | 1 | 1 | 1 | | 1 |
| Q5CUN1 | Protein phosphatase, signal peptide, 2-6 transmembrane domain protein | cgd3_2150 | 28.63 | 1.6152 | 3.2 | 1 | 1 | | 1 |
| Q5CVD8 | Translation initiation factor if-2 betam beta subunit ZnR | cgd8_4430 | 24.274 | 1.6024 | 9.8 | 2 | 2 | | 2 |
| A3FQ11 | Uncharacterized protein | cgd8_5180 | 52.262 | 1.6021 | 2.2 | 1 | 2 | | 1 |
| Q5CRE7 | Uncharacterized protein | cgd5_3080 | 19.482 | 1.5952 | 6 | 1 | 1 | | 1 |
| Q5CRY2 | Apicomplexan conserved protein | cgd5_1030 | 45.391 | 1.5943 | 1.8 | 1 | 1 | | 1 |
| A3FQ58 | Uncharacterized protein | cgd2_910 | 43.601 | 1.5937 | 3.2 | 1 | 1 | | 1 |
| Q5CX16 | Exostosin-like protein | cgd6_2900 | 25.857 | 1.5894 | 5 | 1 | 1 | | 1 |
| Q5CV92 | RecQ4 SF II RNA helicase | cgd8_4950 | 165.96 | 1.5817 | 0.5 | 1 | 1 | | 1 |
| Q5CVN2 | Importin beta like ARM repeat alpha superhelix (Fragment) | cgd8_3440 | 103.66 | 1.5755 | 0.9 | 1 | 1 | | 1 |
| Q7YYX5 | Putative ABC transporter protein, possible | 1MB.836 | 72.336 | 1.5704 | 1.6 | 1 | 1 | | 1 |
| Q7JPJ4 | 15 kDa protein | cp15 | 16.196 | 1.5667 | 4.7 | 1 | 1 | | 1 |
| F0X513 | Cgd5_800 protein | cgd5_800 | 24.186 | 1.5484 | 4.4 | 1 | 1 | | 1 |
| Q5CTP4 | Ribosomal protein L37 (Fragment) | cgd2_2200 | 11.474 | 1.5464 | 6.9 | 1 | 1 | | 1 |
| Q9GZ27 | Multiprotein bridging factor type 1 | MBF1 | 16.448 | 1.5329 | 6.8 | 1 | 1 | | 1 |
| Q7YZ02 | Uncharacterized protein | 1MB.772 | 317.72 | 1.5275 | 0.3 | 1 | 1 | | 1 |
| F0X5T7 | Cgd3_3850 protein | cgd3_3850 | 57.632 | 1.5113 | 4.1 | 2 | 3 | | 2 |
| Q5CV29 | Putative fucose translocator with 8 transmembrane domains, within locus of 3 paralogous genes | cgd3_500 | 42.936 | 1.5108 | 2 | 1 | 2 | | 1 |
| Q7YYI7 | Putative transcription regulatory protein, possible | 1MB.201 | 295.03 | 1.4711 | 0.5 | 1 | 1 | | 1 |
| Q5CW87 | SEC24C-like component of COPII coatamer of ER-golgi vesicles | cgd8_1250 | 127.84 | 1.4201 | 1.4 | 2 | 2 | | 2 |
| Q8T6T2 | Inosine-5'-monophosphate dehydrogenase | 56k.02 | 43.081 | 1.4189 | 3 | 1 | 1 | | 1 |
| F0X3N5 | Cgd4_720 protein | cgd4_720 | 38.849 | 1.4163 | 3.6 | 1 | 1 | | 1 |
| Q7YYT1 | Derlin | 1MB.478 | 31.415 | 1.4015 | 2.5 | 1 | 1 | | 1 |
| Q5CSJ0 | Uncharacterized protein | cgd1_2510 | 32.571 | 1.3788 | 2.4 | 1 | 1 | | 1 |
| Q5CSS2 | Uncharacterized protein | cgd1_1550 | 74.43 | 1.3562 | 2.2 | 1 | 1 | | 1 |
| Q5CYJ4 | WD repeat protein | cgd7_2100 | 260.57 | 1.3521 | 0.7 | 1 | 1 | | 1 |
| Q5CSZ5 | Apicomplexan-conserved protein | cgd1_720 | 24.103 | 1.3479 | 3.3 | 1 | 1 | | 1 |
| Q5CVF6 | FAD/NAD(P)-binding rossman fold oxidoreductase fused to a glucose-methanol-choline (GMC) oxidoreductase domain | cgd8_4230 | 101.27 | 1.3477 | 1.1 | 1 | 1 | | 1 |
| Q5CQN3 | Uncharacterized protein | cgd4_50 | 21.645 | 1.3467 | 3.8 | 1 | 3 | | 1 |
| Q7YYH1 | Uncharacterized protein | 1MB.230 | 31.206 | 1.3445 | 5.6 | 1 | 1 | | 1 |
| Q5CUF7 | TBC domain containing protein | cgd3_3000 | 63.436 | 1.334 | 1.3 | 1 | 1 | | 1 |
| Q5CWB1 | Uncharacterized protein | cgd8_1000 | 44.013 | 1.3068 | 2.8 | 1 | 1 | | 1 |
| Q5CVC6 | Predicted coiled coil protein | cgd8_4580 | 62.382 | 1.3046 | 1.9 | 1 | 1 | | 1 |
| Q5CRI4 | Uncharacterized protein | cgd5_2650 | 15.833 | 1.3045 | 5 | 1 | 1 | | 1 |
| A3FPQ6 | ATPase ASNA1 homolog | cgd7_4070 | 40.885 | 1.2877 | 1.9 | 1 | 1 | | 1 |
| Q7YYJ3 | Uncharacterized protein | 1MB.191 | 23.506 | 1.2546 | 3.4 | 1 | 1 | | 1 |
| A3FPX2 | ATP-binding cassette transporter | cgd6_4700 | 144.43 | 1.2477 | 0.9 | 1 | 1 | | 1 |
| F0X4B4 | Cgd6_3130 protein | cgd6_3130 | 53.699 | 1.2356 | 1.7 | 1 | 1 | | 1 |
| Q5CXG5 | Uncharacterized protein | cgd6_1170 | 28.725 | 1.2283 | 4.3 | 1 | 1 | | 1 |
| Q5CTJ4 | Structural maintenance of chromosomes protein | cgd2_2750 | 155.92 | 1.2198 | 0.7 | 1 | 1 | | 1 |
| Q7YY48 | Uncharacterized protein | 1MB.32 | 16.072 | 1.2137 | 5.2 | 1 | 1 | | 1 |
| Q5CY42 | Oligomeric golgi complex protein 4 | cgd7_3750 | 92.347 | 1.2024 | 1.1 | 1 | 1 | | 1 |
| Q9U565 | Immunodominant 17-kDa antigen (Fragment) | Ag17 | 6.9685 | 1.1982 | 14.1 | 1 | 1 | | 1 |
| Q5CVC7 | Uncharacterized protein | cgd8_4570 | 55.291 | 1.1786 | 1.9 | 1 | 1 | | 1 |
| U5NI94 | 47 kDa protein (Fragment) | CP47 | 12.473 | -2 | 16.1 | 1 | 1 | | 1 |
| Q7YYG7 | Myb proto-oncogene protein, possible | 1MB.236 | 46.741 | -2 | 3.5 | 1 | 1 | | 1 |
| Q7YYA5 | Uncharacterized protein | 1MB.347 | 48.282 | -2 | 3.1 | 1 | 1 | | 1 |
| Q5CX83 | Uncharacterized protein | cgd6_2110 | 111.81 | -2 | 1.1 | 1 | 1 | | 1 |
| Q5CVK9 | Acetyl-CoA carboxylase like biotin dependent carboxylase involved in fatty acid biosynthesis (Fragment) | cgd8_3680 | 307.07 | -2 | 0.3 | 1 | 1 | | 1 |
| Q5CU39 | Dbr1p-like RNA lariat debranching enzyme (Fragment) | cgd3_4330 | 43.155 | -2 | 2.2 | 1 | 3 | | 1 |
| Q5CU36 | Uncharacterized protein | cgd3_4360 | 48.951 | -2 | 1.9 | 1 | 1 | | 1 |
| Q5CTV5 | Possible domain KOG3410, conserved alpha-helical protein | cgd2_1410 | 16.185 | -2 | 7 | 1 | 1 | | 1 |
| Q5CTT3 | ING1 ike protein with an N-terminal globular domain and a PHD domain | cgd2_1670 | 13.583 | -2 | 6.8 | 1 | 1 | | 1 |
| Q5CTT1 | Apicomplexan small protein (Fragment) | cgd2_1690 | 11.264 | -2 | 9 | 1 | 1 | | 1 |
| Q5CTQ8 | Uncharacterized protein | cgd2_2040 | 46.604 | -2 | 2.5 | 1 | 1 | | 1 |
| Q5CTK0 | UDP N-acetylglucosamine transporter-like nucleotide sugar transporter with 10 transmembrane domains | cgd2_2670 | 46.832 | -2 | 2.9 | 1 | 1 | | 1 |
| Q5CTC3 | Uncharacterized protein | cgd2_3590 | 761.01 | -2 | 0.2 | 1 | 1 | | 1 |
| Q5CS36 | Uncharacterized protein | cgd5_380 | 22.902 | -2 | 6.4 | 1 | 1 | | 1 |
| Q5CQX5 | Uncharacterized protein | cgd4_2920 | 70.667 | -2 | 1.5 | 1 | 1 | | 1 |
| Q5CQR2 | Uncharacterized protein | cgd4_3640 | 21.456 | -2 | 5.8 | 1 | 1 | | 1 |
| Q5CQB6 | Ring domain containing protein | cgd4_1360 | 79.274 | -2 | 1.9 | 1 | 1 | | 1 |
| Q5CQ73 | Possible apicomplexan specific, low complexity protein | cgd5_3890 | 37.409 | -2 | 3.1 | 1 | 2 | | 1 |
| Q5CPY9 | Uncharacterized protein | cgd8_270 | 42.984 | -2 | 2.4 | 1 | 1 | | 1 |
| Q5CPX6 | Low complexity coiled coil protein (Fragment) | cgd8_420 | 128.45 | -2 | 0.6 | 1 | 1 | | 1 |
| Q5CPT2 | Possible carboxypeptidase (Fragment) | cgd4_4160 | 51.07 | -2 | 1.5 | 1 | 1 | | 1 |
| G3FIL2 | Glycoprotein 60 (Fragment) | --- | 31.992 | -2 | 6.5 | 2 | 6 | | 1 |
| A3FQR4 | Uncharacterized protein | cgd6_250 | 36.888 | -2 | 2.8 | 1 | 1 | | 1 |
| A3FQR0 | 60S ribosomal protein L29 | cgd2_120 | 7.686 | -2 | 14.7 | 1 | 1 | | 1 |
| A3FQ90 | WD-40 repeat protein, putative | cgd2_2880 | 41.471 | -2 | 2.7 | 1 | 1 | | 1 |
| A0A2H4U2I9 | COWP protein (Fragment) | COWP | 16.94 | -2 | 57.6 | 6 | 54 | | 1 |

MW (kDa) was the molecular weight of each identified protein; Score refered to a simple rule to be used to judge whether a result is significant or not; Coverage (%) was the percent of identified peptdie sequence covering in protein sequence; Peptides were the number of identified peptides; PSMs were described as the number of spectrum which protein matched; Unique peptides indicated number of identified peptides that only come form this protein groups
